# Photon-Driven Cluster Fluxionality: Breaking the Bulk Stoichiometry Ceiling in Subnanometric Molybdenum Oxides

**DOI:** 10.1021/acsnano.6c09355

**Published:** 2026-09-08

**Authors:** Yao Wei, Deborah Perco, Alejandro Santana-Bonilla, Federico Loi, Paolo Lacovig, Silvano Lizzit, Lev Kantorovich, Alessandro Baraldi

**Affiliations:** † Theory and Simulation of Condensed Matter (TSCM), 4616 King’s College London, London WC2R 2LS, U.K.; ‡ Department of Physics, University of Trieste, Via Valerio 2, 34127 Trieste, Italy; ¶ Department of Nanocatalysis, Heyrovský Institute of the Czech Academy of Sciences, Dolejškova 2155/3, 182 00 Prague 8, Czech Republic; ∥ Elettra - Sincrotrone Trieste, S.S. 14 Km 163.5, AREA Science Park, 34149 Basovizza-Trieste, Italy

**Keywords:** fluxionality, nanoclusters, oxidation, molybdenum, oxygen activation, XPS, DFT

## Abstract

We investigate the low-temperature (40 K) oxidation behavior of size-selected molybdenum clusters supported on epitaxial graphene. By combining high-resolution X-ray photoelectron spectroscopy with first-principles density functional theory, we demonstrate that subnanometric clusters can break the stoichiometric ceiling of bulk transition-metal oxides. Quantitative agreement between experiment and theory is achieved only when the clusters accommodate an unusually high oxygen content, with metal-to-oxygen ratios reaching up to Mo:O = 1:5, far exceeding the 1:3 limit of bulk oxide MoO_3_. This superstoichiometric regime is a consequence of coordination plasticity, where the molybdenum framework undergoes pronounced structural expansion to stable tetrahedral MoO_4_ motifs that are energetically inaccessible in extended solids. We provide compelling evidence that this process is mediated by photon-driven structural fluxionality: with kinetic energies exceeding 10 eV, secondary electrons generated during photoemission represent the only source to provide the vibrational excitations necessary to overcome the thermal constraint for structural transformation, allowing the clusters to disrupt the atomic connectivity and reach low-energy isomer configurations characterized by large structural expansion. Furthermore, atomic valence, rather than formal oxidation state, provides a physically meaningful and transferable descriptor for interpreting the main trend in core electron binding energy shifts in nanoscale disordered systems. These findings reveal a fundamentally non-bulk oxidation mechanism governed by non-thermal energy-activated structural dynamics, outlining a strategy for engineering oxygen-rich reactive sites at the ultimate size limit.

## Graphical abstract

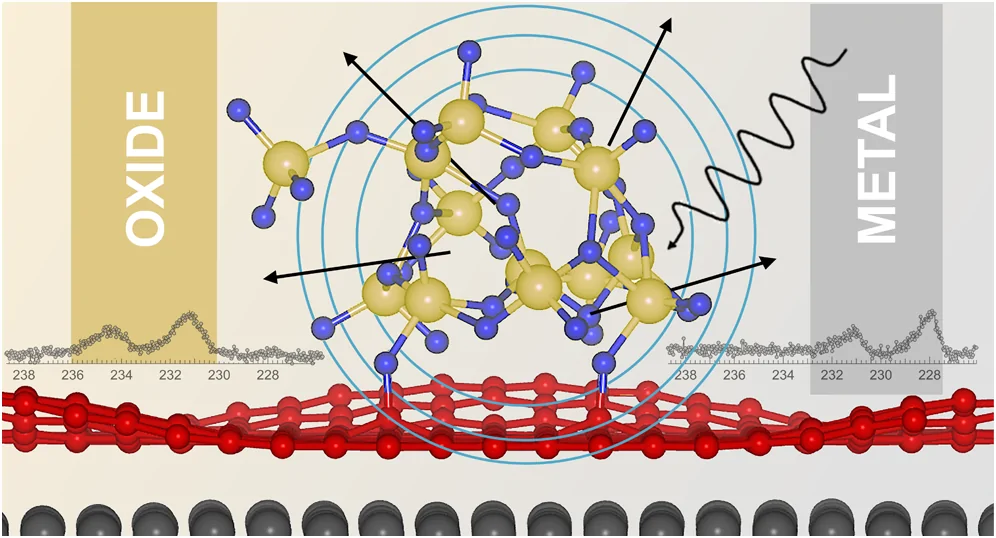

Molybdenum oxides have attracted significant interest in catalysis due to the rich and complex chemistry of this element, which exhibits a wide range of oxidation states, spanning from 1+ to 6+, making it uniquely versatile for a broad spectrum of chemical reactions.^1,2^ The highest oxidation state is realized in the most stable and extensively studied form, molybdenum trioxide (MoO_3_). As an n-type semiconductor, MoO_3_ features a wide bandgap typically ranging from 2.8 to 3.2 eV,^3–6^ although this electronic signature is deeply sensitive to the material’s underlying symmetry. Molybdenum trioxide exhibits three different polymorphs: orthorhombic (α-MoO_3_), monoclinic (β-MoO_3_), and hexagonal (h-MoO_3_),^7,8^ with the first being the most thermodynamically stable. The main building blocks of these compounds are MoO_6_ octahedra, and their different geometric arrangement, whether corner-sharing or edge-sharing, dictates the band structure and specific energy gap of each phase. For instance, while the α and β phases offer tailored transport barriers ideal for hole-injection layers in organic light-emitting diodes (OLEDs),^9,10^ the exposure of specific high-energy crystal facets, such as the (060) facet, can significantly lower oxygen adsorption barriers for advanced gas sensing.^11^ Consequently, MoO_3_ is a cornerstone material in a variety of applications such as desulfurization processes^12,13^ and in the petroleum and chemical industries,^14,15^ or as a promising material for nitrogen oxides removal.^16,17^ It is also used as an electrode material for alkali-ion batteries,^18,19^ owing to its ability to incorporate large quantities of foreign atoms. In addition, when supported on TiO_2_, MoO_3_ also exhibits excellent performance as a photocatalyst.^20,21^

The second-most stable oxide of molybdenum is MoO_2_. The bulk structure of MoO_2_ is isomorphous to that of WO_2_, with which it also shares the oxidation state of the metal cations, namely, 4+.^22^ Although less extensively investigated, molybdenum dioxide, in addition to its applications as a catalyst for partial oxidation of isooctane,^23^ has widely been used as an anode material for lithium-ion batteries.^24,25^

Along with these two stable oxides, molybdenum can form several substoichiometric compounds known as Magneli phases. In these mixed-valence compounds, oxygen deficiency is accommodated through the formation of ordered crystallographic shear planes, which modify the typical framework of corner-sharing MoO_6_ octahedra found in bulk molybdenum oxides.^26,27^ It is noteworthy that, in all molybdenum oxides, Mo atoms are consistently coordinated by 6 oxygen atoms. Among these compounds, the only exception is Mo_4_O_11_, whose structure consists of an alternation of MoO_4_ and MoO_6_ units.

The primary factor distinguishing the different oxides lies in the degree of structural distortion, which is accompanied by a progressive decrease in Mo–O bond lengths as the oxidation state of Mo increases. From this perspective, it must be highly advantageous to investigate oxide nanoclusters specifically, as these could provide a means to generate new bonding geometries that enable fine-tuning of oxidation states and reactivity.^28–30^ Indeed, for example, the presence of terminal metal–oxo groups in WO_3_ clusters is known to play a decisive role in governing their reactivity toward organic molecules.^3,31^ More recently, the synthesis and characterization of ultrasmall nanoparticles have attracted significant attention, as their reduced dimensions induce pronounced structural distortions that can lead to substantial changes in their chemical states, particularly in polar metal oxides such as cerium oxide. For such ultrasmall particles, the intrinsically disordered atomic arrangement is believed to promote structural instability, resulting in the high degree of structural flexibility required to accommodate the large atomic rearrangements associated with oxidation.^32,33^

In this study, we demonstrate that achieving good agreement between experimental and theoretical spectroscopic data for molybdenum oxide clusters requires explicit consideration of the broad distribution of Mo–O bond lengths within the clusters and approximating the oxidation state of Mo atoms with their valence, as defined within the Bond Valence Model.^34^ Employing this valence framework enables a more consistent interpretation of the Mo 3d HR-XPS (high-resolution X-ray photoelectron spectroscopy) spectra and allows for an accurate assignment of oxidation states in these complex systems. Most importantly, we have found that the experimental findings can be explained only by taking into account the formation of clusters with exceptionally high oxygen densities (as schematically represented in Figure 1), which greatly exceed the expected Mo:O ratio of 1:3 found in the bulk Mo oxide. Notably, clusters are deposited and oxidized at only 40 K, where the thermal energy is insufficient for large structural rearrangements or oxygen incorporation beyond bulk limits. Taken together, our experimental observations and DFT calculations strongly support a mechanism in which secondary electrons produced in the photoemission process not only induce O_2_ dissociation but also, more importantly, provide the vibrational excitation necessary to activate structural fluxionality. This mechanism enables the clusters to overcome kinetic barriers even at 40 K, thereby promoting structural rearrangements among low-lying isomers and allowing the accommodation of oxygen densities far exceeding the bulk Mo:O stoichiometry.

**Figure 1:**
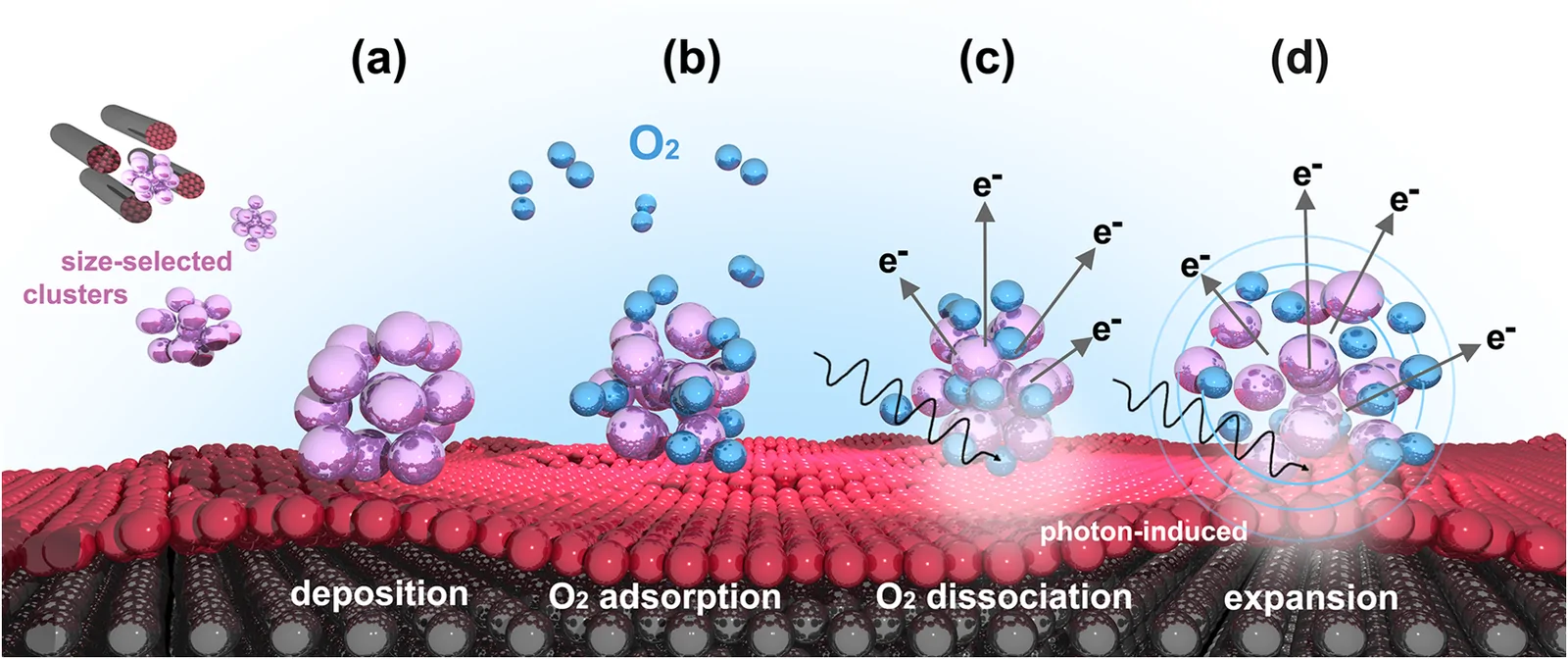
Schematic representation of our experiment. (a) Soft-landing deposition of size-selected Mo clusters on graphene epitaxially grown on Ru(0001); (b) O_2_ exposure at 40 K; (c) soft X-ray irradiation and photon-induced dissociation of molecular oxygen; (d) cluster expansion and oxygen accumulation.

## Results and Discussion

Size-selected Mo^+^ clusters with 6 and 13 atoms were generated by means of ENAC,^35^ and a coverage of 0.03 % ML was deposited in situ at 40 K onto graphene epitaxially grown on Ru(0001) (see the Experimental Methods section). The low deposition temperature helps to prevent cluster diffusion and sintering, which are crucial for preserving mass selection. Mo_6_ and Mo_13_ were chosen as the model clusters for this study. Mo_13_ is a well-known magic-number cluster for molybdenum, whereas Mo_6_ was included due to predictions of its high reactivity.^36^ Mo 3d core-level spectra (spin-orbit splitting of 3.1 eV) of the pristine clusters were acquired at a photon energy of 320 eV and are shown in Figure 2 after background subtraction (see the Experimental Methods section). An initial analysis of the Mo 3d spectra revealed distinct differences between the two clusters. For Mo_6_, the 3d_5/2_ main peak, located at 228.1 eV (black dotted line), is shifted by 0.2 eV relative to Mo_13_, with both clusters exhibiting BE comparable to, or slightly higher than, that of bulk metallic molybdenum (227.9 eV), consistent with their metallic character.

**Figure 2:**
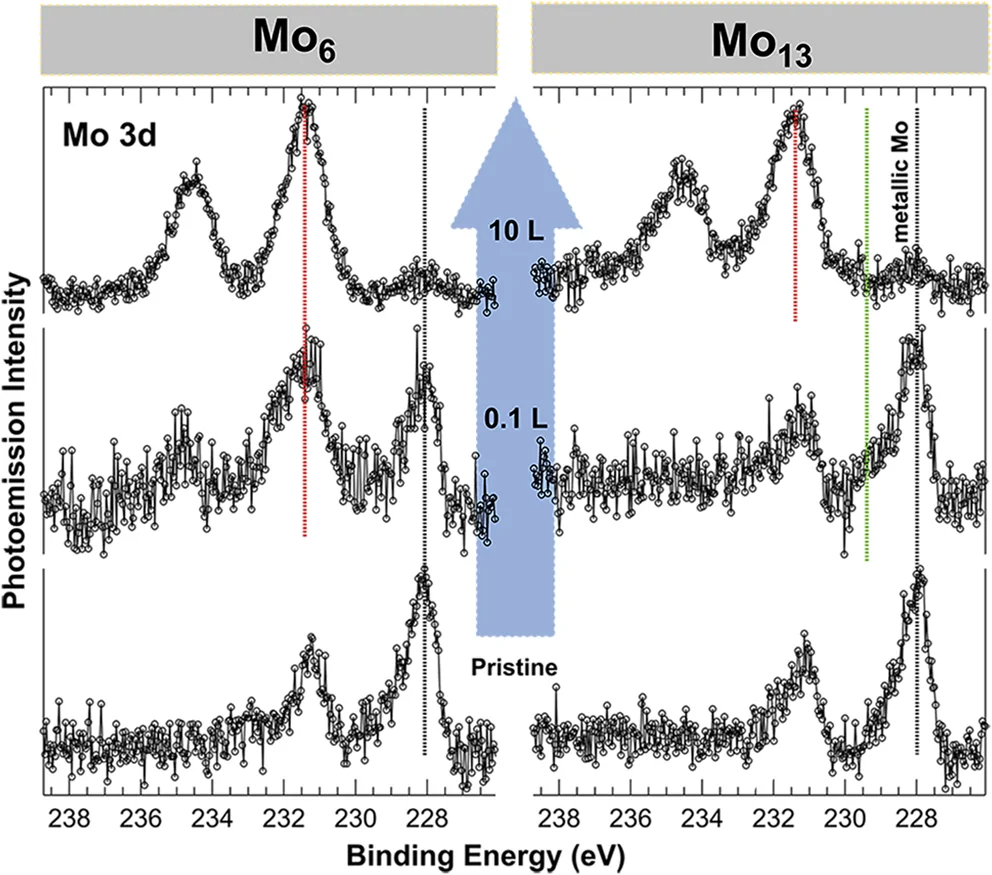
Mo 3d spectra for both Mo_6_ (left) and Mo_13_ (right) clusters acquired before oxygen exposure (bottom) and after 0.1 L (middle) and 10 L oxygen exposure (top) (both systems were irradiated for 20 min with X-rays at 320 eV). Black, green, and red dotted lines are used as a visual guide for the various Mo 3d_5/2_ components, metallic and oxidized ones.

Following deposition, the Mo clusters were exposed to molecular oxygen pressure in two steps: the first exposure of 0.1 L (*p*_O2_ = 1 × 10^–9^ mbar, 133 s) and the second of 10 L (*p*_O2_ = 5 × 10^–8^ mbar, 266 s), both while being irradiated with soft X-rays. The cluster was further irradiated with 320 eV energy photons for a total of 20 min. The effectiveness of this strategy in breaking the O_2_ bond and producing atomic oxygen species, which has already been demonstrated in previous studies,^37–39^ is also confirmed for this case of molybdenum clusters on graphene, as shown in Figure S1 of the Supporting Information, where the Mo 3d core-level spectra show a clear difference depending on whether the region was irradiated for prolonged time with soft X-rays or not.

Mo 3d core-level spectra acquired after Mo_6_ and Mo_13_ exposure to different doses of oxygen are reported in Figure 2 for the two different exposures. Upon oxidation and irradiation, a positive shift of the main spectral components toward higher BE is observed, in line with expectations. After 0.1 L exposure, the spectra of both clusters broaden significantly. A key difference between the two clusters emerges at this stage: Mo_6_ displays a new component at much higher BE, centered approximately at 231.4 eV for the 3d_5/2_ peak (red dotted line), consistent with Mo atoms in the 5+ oxidation state. In contrast, Mo_13_ exhibits, in addition, a lower BE component centered at 229.8 eV for the 3d_5/2_ peak, corresponding to the 4+ oxidation state (green dotted line). After an even larger oxygen exposure (10 L), the two spectra become quite similar, with only a small fraction of metallic Mo remaining while the dominant spectral feature is the 231.4 eV component.

Due to the complexity of assigning oxidation states to specific components, as shown in studies of W clusters,^40^ DFT calculations were performed to support data interpretation.

Recent ab initio DFT simulations based on a random structure searching technique provided the lowest-energy configuration of Mo_6_ on free-standing graphene;^41^ this cluster was then placed in the FCC valley region of the graphene moiré, where the carbon-Ru distance is the shortest, and relaxed. For the Mo_13_ cluster, the random structure searching AIRSS method^42–44^ was first used to determine the lowest-energy gas-phase Mo_13_^+^ cluster and then to explore different orientations on graphene/Ru(0001) to identify the most stable geometry. As evidenced from Figure 3(a), Mo_6_ adopts a double-layer structure consisting of two triangular units stacked with a 180° rotational offset. In contrast, Mo_13_ exhibits a more disordered geometry, with atoms not forming distinct planes (Figure 3(b)).

**Figure 3:**
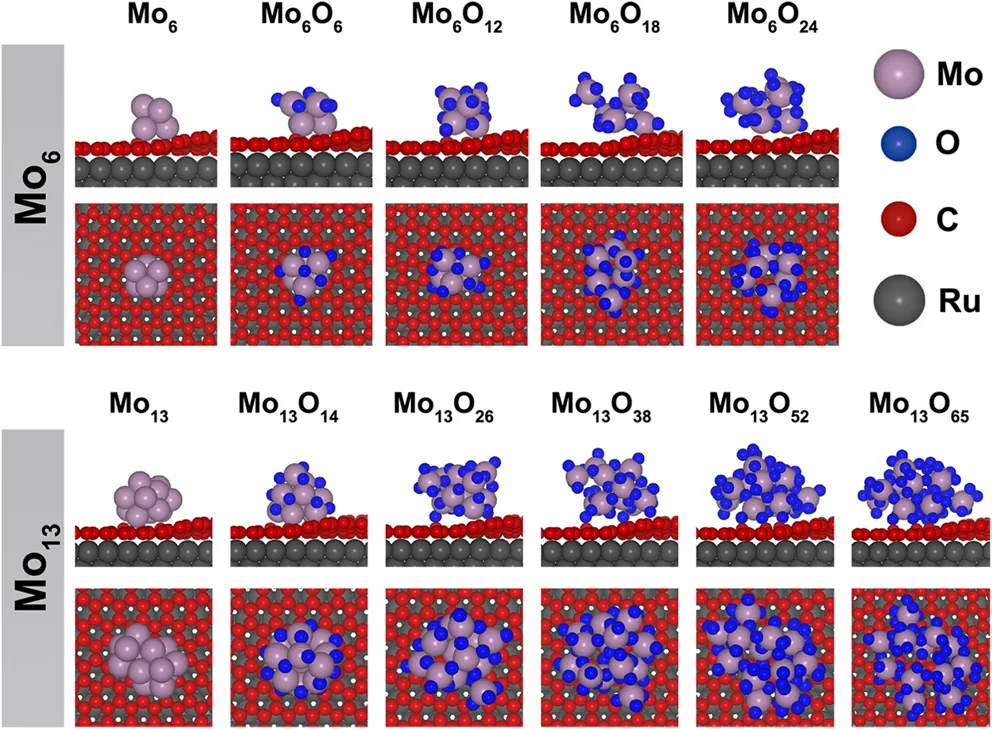
Top and side views of relaxed geometries of pristine and selected oxidized (a) Mo_6_ and (b) Mo_13_ clusters. Ru, C, Mo and O atoms are depicted in gray, red, pink and blue colors, respectively.

Initially, to model Mo cluster oxidation, oxygen atoms were randomly placed in pairs (because of molecular dissociation), exploring all the Mo_6_O_2*x*_ (*x* = 1–9) and Mo_13_O_2*x*_ (*x* = 1–19) stoichiometries, achieving the typical Mo:O ratio of 1:3 of bulk Mo oxides.

However, as it will become clear later, these oxygen densities were found insufficient to explain experimental spectra. Therefore, higher oxygen densities were additionally investigated by considering Mo_6_O_24_, Mo_13_O_52_, and Mo_13_O_65_ cluster configurations. Representative relaxed geometries of oxidized clusters are reported in Figure 3(a) (Mo_6_) and (b) (Mo_13_) for selected Mo:O ratios of about 1:1, 1:2, 1:3, 1:4, and 1:5. In addition to the lowest energy structure of Mo_13_O_65_ shown in Figure 3(b), three additional relaxed isomers (C1, C2, and C3) lying 0.13, 0.27, and 4.03 eV higher in energy were examined (see Figure S2 of the Supporting Information) and included in our analysis.

The O–O bond-length distributions calculated for Mo_13_O*_n_* (*n* = 14, 26, 38, 52 and 65) clusters (see Figure S3 of the Supporting Information) suggest that peroxide- and superoxide-like species can be present only in negligible amounts. Indeed, only at the highest oxygen coverages does a small fraction of O–O distances fall within the 1.3–1.4 Å range characteristic of these species, suggesting the presence of residual peroxide/superoxide species. Nevertheless, these species account for less than 1% of the oxygen distances in the simulated nanoclusters.

Furthermore, DFT calculations reveal an oxygen coverage-dependent oxidation mechanism coupled to the structural expansion of the Mo clusters. Oxygen incorporation into the cluster interior becomes thermodynamically favorable for Mo_13_ only above a critical surface coverage of approximately 30 oxygen atoms and requires overcoming the energy barriers of about 3–4 eV (see Figure S4 of the Supporting Information). At lower O densities, the lowest energy configurations correspond to oxygens remaining confined to the cluster surface.

What clearly emerges is a structural expansion driven by coordination plasticity: as the oxygen density rises, the molybdenum framework locally expands, overcoming the steric hindrance that typically constrains oxygen incorporation in rigid bulk lattices. The evolution of the Mo–Mo bond-length distribution, reported in Figure S5 of the Supporting Information, clearly shows that this quantity increases with oxygen coverage, evolving from values comparable to the nearest-neighbor distance in bulk molybdenum (2.74 Å) to values that even exceed the second nearest-neighbor distance (3.17 Å) there. Interestingly, this expansion is more pronounced in oxidized Mo_6_ than in oxidized Mo_13_ (see Figure S6 in the Supporting Information), indicating that the smaller cluster undergoes a larger structural distortion upon oxidation.

For each atomic configuration, Mo 3d_5/2_ core levels were computed for selected Mo:O ratios (see Figure 4(a)), for a total of 147 values. Notably, none of the core-level energies calculated for the Mo_*y*_O_3*y*_ configurations (*y* = 6, 13), corresponding to the maximum metal-to-oxygen ratio characteristic of bulk molybdenum oxides, reproduce the dominant 3d_5/2_ component observed experimentally after the 10 L O_2_ exposure and X-ray irradiation. In particular, the experimental BEs are systematically higher than those predicted for stoichiometric Mo oxide clusters. To achieve quantitative agreement with the experimental spectra, markedly oxygen-rich configurations must be considered.

**Figure 4:**
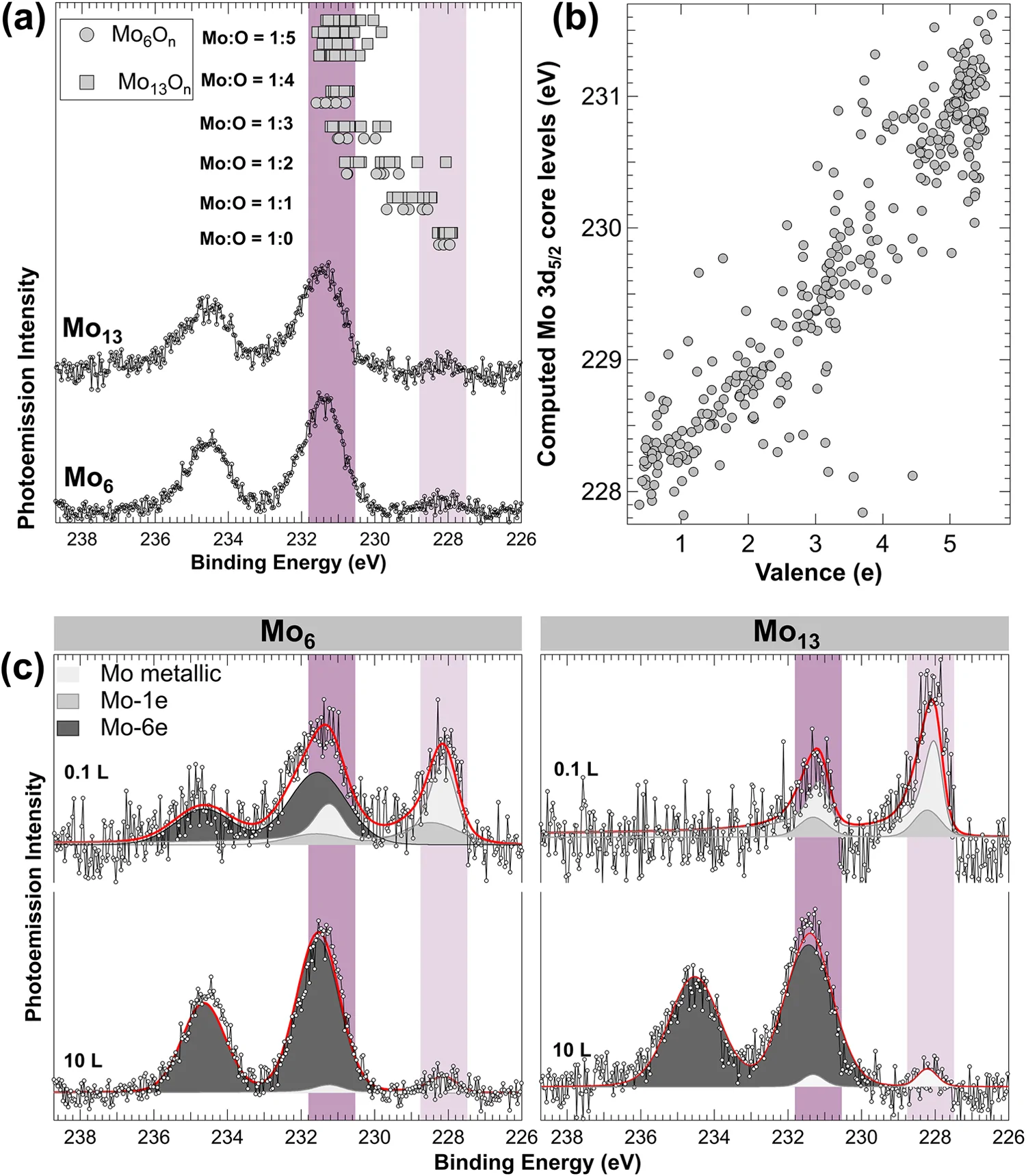
(a) Measured 3d core-level spectra for Mo_6_ and Mo_13_ clusters (bottom) and computed 3d_5/2_ core levels for selected oxidized Mo clusters having different Mo:O ratios (top). (b) Dependence of the computed 3d_5/2_ core levels on the Mo valencies. (c) Fits of Mo 3d core level spectra for oxidized Mo_6_ and Mo_13_ clusters using the valence approach. The white component is associated with the metallic Mo atoms, the light gray one with Mo-1e family, and the dark gray one with Mo-6e family. Light and dark violet rectangles are used as a visual guide for 1:0 and 1:5 Mo:O ratios, respectively.

In particular, a Mo:O ratio of 1:4 is required for Mo_6_, while an even higher oxygen content, corresponding to a Mo:O ratio of 1:5, is necessary for Mo_13_.

These findings are supported by the calculations of Mo 3d core-level BEs for isomers of the Mo_13_O_65_ cluster, which have exhibited very similar distributions across all four configurations (Figure 4(a)), thus supporting the conclusion that the associated core-level spectra are very similar.

These stoichiometries substantially exceed those typical of bulk molybdenum oxides, indicating that Mo clusters can accommodate an unexpectedly large amount of oxygen within their structures. This finding points toward a fundamentally different oxidation behavior at the subnanometric scale, which cannot be inferred from the bulk stoichiometries alone.

Considering that Mo bulk oxides mainly differ in crystal structure and Mo–O bond lengths, rather than in the Mo–O coordination number, which is equal to 6, Mo 3d core-level spectra were analyzed using a model based on metal–oxygen coordination and bond lengths, expressed in terms of the atomic valences.^45,46^ Here, the valence is defined according to the Bond Valence Model as the sum of the individual bond valences associated with a given atom due to all its nearest neighbors, a quantity known to strongly influence core-level BE shifts. This approach has previously been shown by Shimoda *et al.* ^47^ to be effective in reproducing the Mo 3d core-level spectrum of Mo_4_O_11_, a compound characterized by the presence of four crystallographically non-equivalent Mo sites in the lattice. The calculated valences as a function of the corresponding computed core-level BEs are reported in Figure 4(b), demonstrating a good correlation. Assuming that the atomic valence provides a good approximation of the oxidation state,^46^ Mo atoms can be grouped into six distinct families, each centered around an integer valence value, s, and labeled Mo-1e (centered at s = 1), Mo-2e (s = 2), and so forth, up to Mo-6e (s = 6). Based on this classification, we constructed the spectral line shape associated with each family and used them to fit the experimental Mo 3d spectra of oxidized molybdenum clusters (see the Experimental Methods section for a more comprehensive explanation of the fitting procedure). As shown in Figure 4(c), the resulting agreement between theoretical predictions and the experimental data fit is remarkably good and supports our interpretation. This method accurately reproduces the values of the high BE components, which are especially sensitive to oxidation effects. These results demonstrate that atomic valence can be used as a descriptor that captures the local coordination environment more accurately than formal oxidation states, providing a consistent framework to map the electronic evolution of fluxional, disordered nanosystems. It is important to emphasize that, although there is a clear relationship between core-level shift and valence, the bond valence model does not account for important effects that can contribute to the experimentally measured shifts, among which certainly is the contribution of final-state effects such as the screening of valence electrons upon the creation of a core-hole.

The unusually large number of oxygen atoms accommodated in Mo clusters can be rationalized by the specific way in which oxygen atoms arrange within the clusters’ framework. To this end, the coordination of O by Mo atoms has been analyzed, as well as the coordination of Mo by O atoms in all clusters as a function of the O density and the Bader charge. Starting from the latter, which is reported in Figure S7 of the Supporting Information, the general trend is that Mo atoms donate, as expected, their electrons to the nearest oxygens, with the amount of charge being crucially dependent on the Mo coordination with oxygens: the highest donation (up to 2.5e) is found for Mo atoms with coordination above five. As the O density increases, a larger fraction of Mo atoms becomes highly coordinated, and the distribution of the donated charge progressively narrows, saturating at approximately 2.5e. This value is in excellent agreement with the reported range of 2.35–2.68e for Mo in crystalline Mo oxide.^48,49^ In contrast, oxygen atoms show the opposite trend. At low O densities, all O atoms gain about −1e from neighboring Mo atoms. At higher O densities, however, the charge distribution broadens significantly, ranging from −0.23e to −1.11e, reflecting the flexible, noncrystalline topology discussed above.

To assess whether the interaction with the substrate also plays a role in determining the properties of the Mo clusters, we performed a charge density difference calculation for the Mo_13_O_2_ cluster supported on both graphene/Ru(0001) and graphene only. As shown in Figure S8 in the Supporting Information, there is a clear charge transfer from the Mo cluster to the graphene layer. In contrast, the contribution of the Ru substrate is relatively small, leading to only a partial compensation of the electron withdrawal induced by graphene. Moreover, the charge transfer strongly depends on the distance between the metal atoms and the substrate: the electron depletion is significantly more pronounced for the bottom-layer Mo atoms, i.e., those directly facing the graphene (which becomes slightly n-doped), than for the atoms located in the upper part of the cluster. The observed charge density difference, concentrated at the interfacial Mo atoms, points to a depletion of electron density that could weaken the local Mo–Mo bonding. However, it is important to notice that the carbon–molybdenum distance increases as a function of the oxygen density. This points toward a reduced interaction with the substrate as clusters are oxidized, due to the stronger interaction with O atoms. The substantial Mo to O charge transfer strongly affects the Mo 3d core electron BEs. Focusing on Mo atoms coordinated to 4, 5, and 6 O atoms, Figure 5(a) shows an inverse correlation between the average Mo–O bond length and the computed core levels: shorter bonds correspond to higher BEs. In all cases, Mo atoms reach a maximum Bader charge of 2.5e, consistent with the Mo^6+^ oxidation state. Notably, some four-fold coordinated Mo atoms exhibit significantly shorter bond lengths than those with higher coordination.

**Figure 5:**
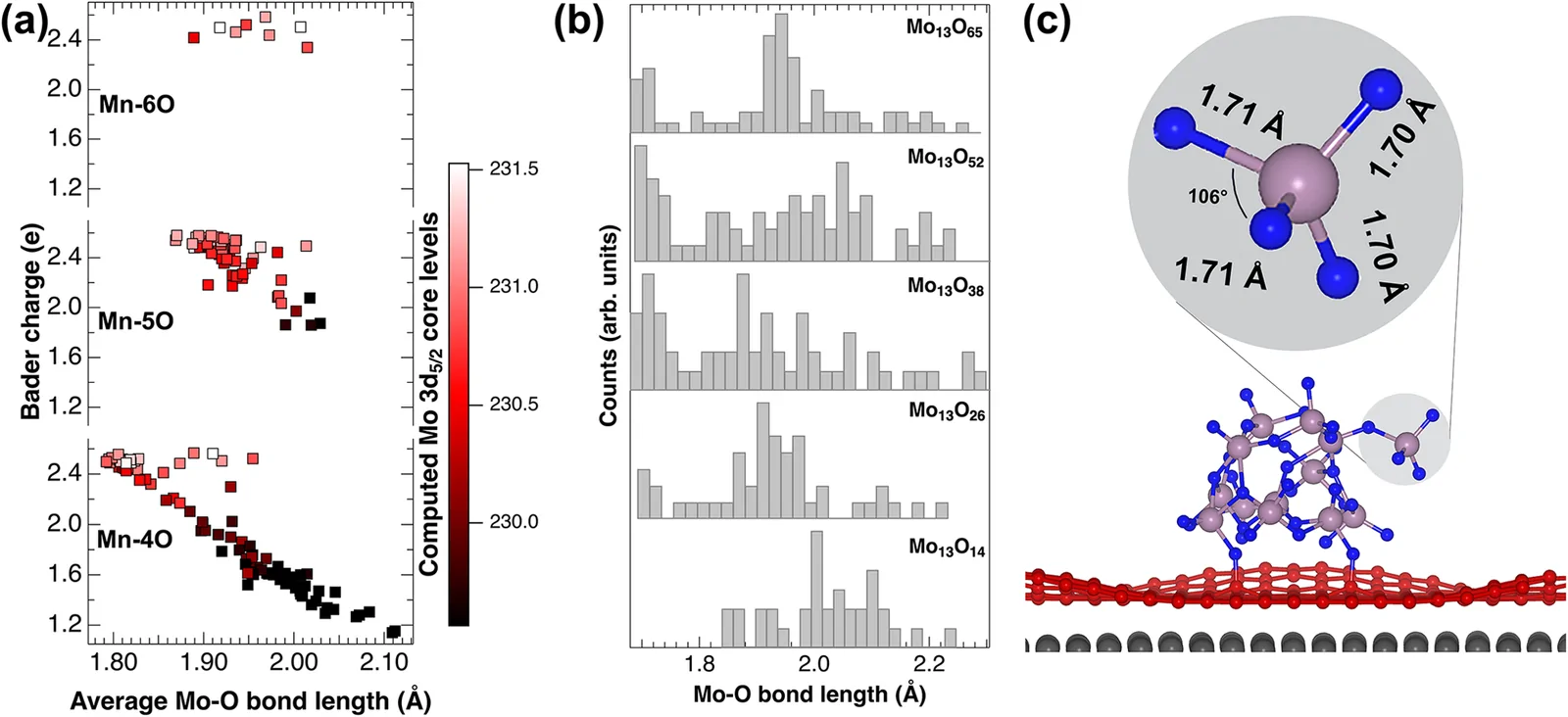
(a) Bader charge of Mo atoms coordinated to 4, 5, and 6 oxygen atoms as a function of the average Mo–O bond lengths. Colors are used to depict the corresponding computed Mo 3d_5/2_ core levels. (b) Mo–O bond length distribution for different Mo:O ratios for Mo_13_. (c) Structural tetrahedral MoO_4_ motifs forming at high oxygen densities.

To further investigate the trend, we computed the Mo–O bond length distribution for Mo_13_ clusters with Mo:O ratios equal to 1:1, 1:2, 1:3, and 1:4.

As shown in Figure 5(b), for high Mo:O ratios (low oxygen content), the distribution is centered around 1.9–2.1 Å. As the ratio (the oxygen content) decreases (increases), not only does the center of the distribution shift toward shorter bond lengths, but a prominent peak near 1.7 Å emerges already for Mo_13_O_14_ and becomes dominant as the oxygen content increases. These shorter bond lengths are indicative of Mo=O double bonds, which are associated with the formation of terminal MoO_4_ units in the clusters. Representative examples of these MoO_4_ units are illustrated in Figure 5(c). To further analyze this behavior, the Mo–O coordination was examined in detail. As shown in Figure 6(a), for Mo_6_O_2*x*_ clusters (upper panel), oxygen atoms at low oxygen densities are predominantly coordinated to 2 or 3 Mo atoms, consistent with bulk-like bridging oxygen environments. As the oxygen content increases, the fraction of singly coordinated oxygen atoms (O-1Mo) rises steadily and becomes dominant at high oxygen densities. This trend indicates the progressive formation of terminal oxygen species, accompanying the pronounced structural expansion of the small cluster. At the same time, the decrease in threefold-coordinated oxygen atoms highlights the limited ability of the small cluster to maintain highly connected oxide networks at high oxygen densities.

**Figure 6:**
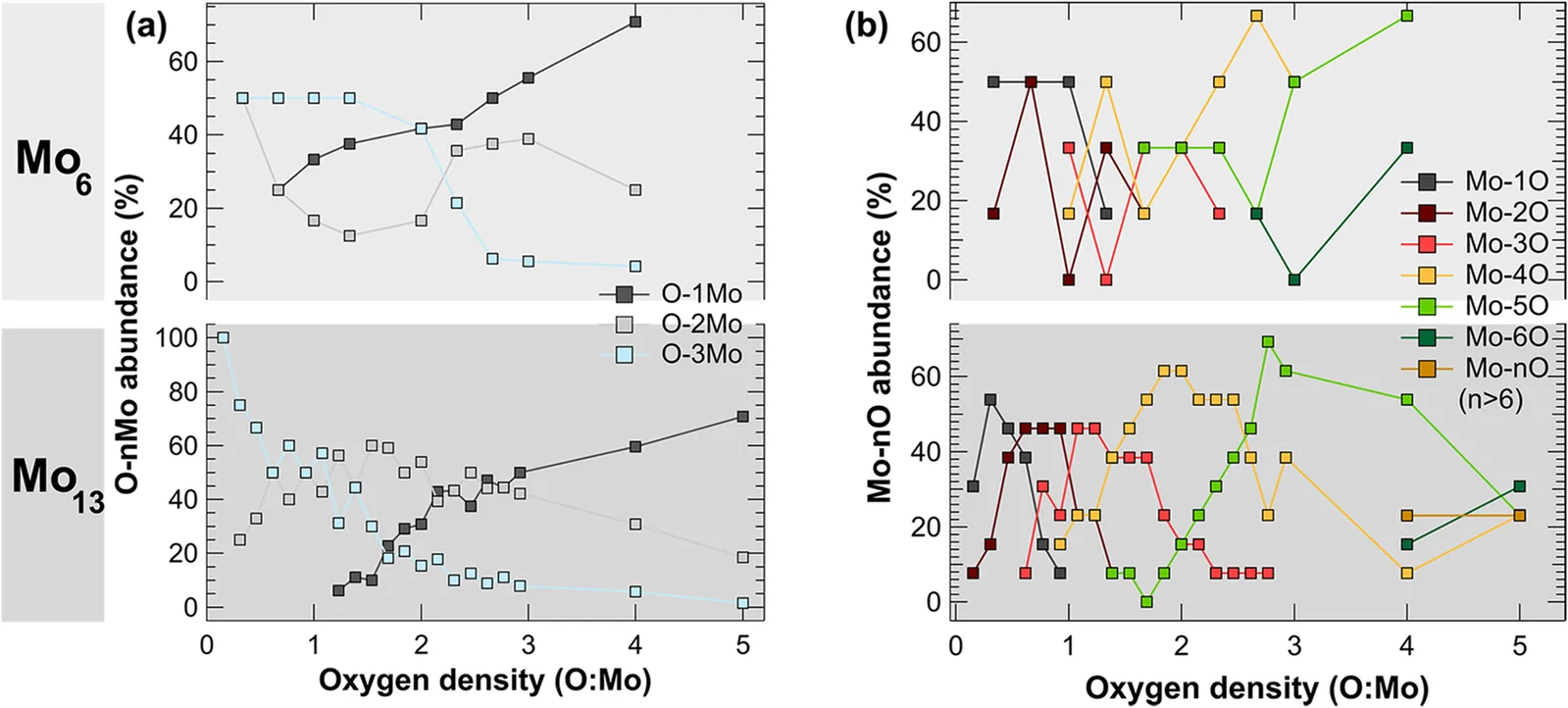
(a) Percentage of O atoms bonded to n-Mo atoms as a function of the oxygen density in oxidized Mo_6_ (upper panel) and Mo_13_ (lower panel). (b) Percentage of Mo atoms bonded to n-O atoms as a function of the oxygen density in oxidized Mo_6_ (upper light gray panel) and Mo_13_ (lower dark gray panel).

In contrast, Mo_13_O_2*x*_ clusters (lower panel) retain a significant fraction of multiply coordinated oxygen atoms over a much wider range of oxygen densities. At low oxygen contents, most O atoms are coordinated to three Mo atoms, consistent with bulk-like oxide motifs. Only with increasing oxygen density, the population gradually shifts toward twofold and eventually singly coordinated oxygen atoms, although this transition occurs at much higher oxygen contents than in Mo_6_O_2*x*_ clusters. This delayed appearance of O-1Mo species demonstrates the enhanced capacity of the larger cluster to distribute oxygen atoms over multiple Mo centers. At the highest oxygen densities, a large fraction of oxygen atoms becomes singly coordinated, consistent with the formation of oxo ligands and peripheral oxygen species. In agreement with this, the Mo_13_ core-level spectrum after high O_2_ exposure cannot be reproduced using a Mo:O ratio of only 1:4, indicating that a critical fraction of singly coordinated oxygen atoms, approximately 70%, is present. These atoms form terminal Mo=O double bonds, further supported by the significantly shorter Mo–O bond length (Figure S9 in the Supporting Information). Although such tetrahedral coordination is rare in bulk molybdenum oxides, occurring only in Mo_4_O_11_ within a mixed MoO_4_–MoO_6_ lattice, it is a common and electronically stable motif in molecular systems.

As the oxygen density increases, the coordination number of Mo with O atoms increases accordingly, as shown in Figure 6(b) for both families of clusters. For Mo_6_O_2*x*_ (top panel), the oxidation process is characterized by a rapid evolution of Mo coordination environments. At low oxygen densities, Mo atoms are predominantly low-coordinated to O, with a significant fraction having one or two oxygen neighbors. As the oxygen content increases, there is a swift transition toward higher coordination numbers. Notably, tetrahedrally coordinated Mo atoms (Mo-4O) become dominant over a relatively narrow range of oxygen densities, highlighting the strong structural response of the small cluster to oxidation. At the highest oxygen densities, a substantial fraction of Mo atoms reaches a coordination of 5 and 6, indicating the formation of highly oxidized local environments within the cluster. Mo_13_O_2*x*_ clusters (bottom panel) display a more gradual and extended evolution of the Mo coordination. At low oxygen densities, a broad distribution of coordination numbers is already present, reflecting the larger size and structural heterogeneity of the clusters. As oxidation proceeds, the fraction of Mo atoms with intermediate coordination (three to four oxygen neighbors) increases steadily over a wide range of oxygen contents. Only at higher oxygen densities does a significant population of highly coordinated Mo atoms (five to seven oxygen neighbors) emerge, demonstrating that the larger clusters can accommodate additional oxygen. This difference underpins the distinct charge-transfer behavior reported in Figure S7 and explains why Mo_13_ clusters can sustain higher oxygen densities before reaching charge saturation.

A key question arises at this point: at low temperature (40 K), where thermal energy is negligible, what provides the activation energy for the observed cluster expansion and structural modifications that accommodate excess oxygen (Mo:O up to 1:5)? Our results demonstrate that secondary electrons generated during the photoexcitation process play a dual role. As well as inducing O_2_ dissociation by promoting electrons from bonding to antibonding molecular orbitals^37–39^ (the calculated energy barrier for a dissociation process not based on electronic excitations was found to be between 0.2 and 0.3 eV in the case of Mo_13_ which is too high for the low temperature (40 K) used by us, see Figure S10 in Supporting Information), low-energy electrons with kinetic energies of only a few eV, but far exceeding the thermal distribution, can also provide enough energy to overcome the cluster restructuring, in a similar way hot electrons do for plasmonic excitations,^51^ which are known to play a vital role in catalysis.^52^

The dissipated energy can cause structural fluxionality in Mo clusters, thus facilitating the interconversion among isomers with similar stability but distinct morphologies and the incorporation of O in sites that would usually be hindered in the rigid structure. The concept of fluxionality represents the hallmark of subnanometer clusters lacking bulk rigidity and has emerged as a key factor in understanding nanocluster activity.^53–55^ For instance, multiple metastable isomers have been shown to contribute to the hydrogen evolution reaction in supported Pt nanoclusters.^56^ Under reaction conditions, clusters can undergo transitions between distinct metastable geometries (plastic fluxionality) and experience significant deformations around their equilibrium structures (elastic fluxionality), particularly in the presence of adsorbates.^57^ A notable example is the oxidation of Cu clusters, where oxygen atoms incorporate into the cluster rather than remain adsorbed on the surface. The energy released along the reaction pathway, together with thermal vibronic excitations, promotes the formation of a variety of isomers; consequently, processes such as O_2_ dissociation can proceed via multiple pathways, leading to diverse oxidized structures.^58^ In our case, for pristine Mo_6_ and Mo_13_, DFT reveals multiple low-energy isomers differing by less than 2 eV (Figure S11), barriers easily bridged by secondary electron impacts. This enables phononic softening, Mo–Mo bond elongation (as reported in S5), and creation of space for terminal O insertion, forming stable MoO_4_ tetrahedral units despite low T. Thus, photoemission not only probes but also actively drives oxidation via photoelectrons, explaining the anomalously high O uptake.

Eventually, the emergence of the tetrahedrally coordinated Mo centers at moderately high oxygen densities establishes a direct link between the oxidation process of molybdenum clusters and that observed in molecular and low-dimensional systems. Tetrahedral MoO_4_ motifs are well known to be intrinsically electronically stable in molecular complexes ([MoO_4_]^2–^ molybdate anion)^59,60^ and isolated polyoxometalate units ([Mo_7_O_24_]^6–^, [Mo_12_O_40_]^*n*−^ Keggin-type units),^61^ where high oxygen coordination is stabilized by terminal oxo ligands in the absence of extended lattice constraints. In contrast, their occurrence in extended bulk oxides is strongly limited by the requirement of the lattice coordination, which is known to typically favor octahedral configurations.

At the nanoscale, molybdenum clusters can effectively overcome these bulk constraints by adopting bonding topologies dominated by terminal oxo species and highly coordinated metal centers with up to seven O atoms surrounding Mo atoms. This structural flexibility enables the stabilization of an oxygen density that significantly exceeds the stoichiometric limits of bulk molybdenum oxides. The ability to accommodate these variable oxygen environments, some presenting molecular-like motifs, thus provides an atomic-scale rationale for the unexpectedly high oxygen densities observed in oxidized Mo clusters and highlights the fundamentally different nature of oxidation processes in subnanometric systems compared to extended solids.

## Conclusions

Our combined experimental and theoretical investigation reveals that subnanometric Mo clusters escape the thermodynamic constraints governing bulk oxides, instead exhibiting a distinctive superstoichiometric oxygen uptake. By reaching Mo:O ratios as high as 1:5, these clusters occupy a previously uncharted compositional regime that bridges crystalline solid-state oxides with molecular polyoxometalates.

We introduce the concept of photon-driven fluxionality to explain how sub-nanometric matter can undergo extensive structural rearrangements even at cryogenic temperatures (40 K): secondary electrons generated during soft X-ray irradiation supply the energy needed to drive the system toward highly oxidized configurations. The intrinsic amorphousness of these clusters further enables the formation of terminal Mo–O bonds and tetrahedral coordination motifs that are sterically inaccessible in rigid, translationally symmetric bulk lattices. This mechanism fundamentally extends prior notions of cluster fluxionality, whether thermal, i.e., arising from small energy differences between degenerate configurations, or reaction-induced, by demonstrating that electronic excitation alone can unlock otherwise kinetically forbidden oxidation pathways.

Finally, by validating the bond valence model as a reliable descriptor for core-level BE shifts in disordered Mo oxides, we establish a transferable framework for characterizing fluxional, non-crystalline nanomaterials with standard X-ray photoelectron spectroscopy.

More generally, our results suggest that photon-induced fluxionality is not intrinsically linked to X-ray excitation but rather to the generation of sufficiently energetic secondary electrons capable of activating molecular species (such as O_2_) and cluster vibrational modes. This concept could therefore be extended to alternative excitation schemes based on extreme ultraviolet (EUV) radiation. Recent advances in compact EUV sources, including plasma-based emission schemes and high-harmonic-generation approaches, provide new opportunities for generating energetic photons outside synchrotron radiation facilities. Such laboratory-scale sources could potentially enable secondary-electron-driven activation processes under more accessible experimental conditions, opening new perspectives for modifying the structure and chemical reactivity of subnanometric clusters.

## Methods

### Experimental Methods

The Ru(0001) crystal was cleaned by cycles of Ar^+^ sputtering and annealing up to 1500 K in an O_2_ atmosphere. The residual oxygen was finally removed by a flash annealing in vacuum to 1500 K. Graphene was grown by chemical vapor deposition of ethylene (C_2_H_4_) at 1000 K in two stages: the first one with a pressure of 2 × 10^–8^ mbar, the second one with an increased pressure of 5 × 10^–8^ mbar to guarantee the complete covering of the surface.^62–64^

Mo_6_^+^ and Mo_13_^+^ were generated by means of the laser ablation cluster source ENAC (Exact Number of Atoms in Each Cluster), where the mass selection is performed by means of a quadrupole mass analyzer and is monitored by acquiring mass spectra (see Figure S12 in the Supporting Information). Size-selected molybdenum clusters were deposited directly in situ on graphene/Ru(0001), on which they are electrically neutralized, in soft-landing conditions, i.e., with a kinetic energy less than 1 eV/atom.^65^ The deposited cluster coverage was 0.06% ML (1 ML = 1.56 × 10^15^ atoms/cm^2^), and it was monitored by integrating the measured cluster current on the sample. During depositions, oxidation, and XPS measurements, the temperature of the sample was kept below 40 K to avoid cluster diffusion and nucleation, thus preserving the size selection.

Clean deposition conditions were ensured by maintaining the partial pressures of H_2_O and H_2_ below 1 × 10^–10^ mbar throughout the experiments. In addition, the cleanliness of the deposition environment, particularly with respect to H_2_O, OH, and H_2_ contamination, was verified by exposing a clean Ir(111) crystal to the same deposition conditions (temperature and exposure time) used for the cluster experiments. Subsequent temperature-programmed desorption (TPD) measurements confirmed that the signals associated with these species remained below the detection limit of our experimental setup (0.5% ML).

High-resolution X-ray photoelectron spectroscopy (HR-XPS) measurements were performed at the SuperESCA beamline at the Elettra synchrotron radiation facility in Trieste, Italy. The core-level spectra were collected by means of a Phoibos-SPECS hemispherical electron energy analyzer (150 mm mean radius) equipped with a delay line detector. The overall energy resolution was better than 80 meV for the photon energies and acquisition parameters employed. During data acquisition, the pressure in the UHV systems was always below 2×10^‑10^ mbar, thus preventing the adsorption of impurities. The XPS spectra were acquired at normal emission and by tuning the photon energy to enhance the surface sensitivity (photoelectron kinetic energy of about 100 eV). For each spectrum, the photoemission intensity was normalized to the photon flux and the BE scale was aligned to the Fermi energy of the Ru substrate. Mo 3d core-level spectra of as-deposited clusters were fitted using a convolution between Doniach–Sunjic line shape^66^ and a Gaussian line shape to account for experimental, inhomogeneous, and phonon broadening.^67,68^ The Lorentzian width values for the two nanoclusters are very similar, being in the range 0.08–0.13 eV for both 3d_5/2_ and 3d_3/2_ spin-orbit splitting components. Notably, the asymmetry of Mo_13_ is significantly lower, i.e., 0.06, than that of Mo_6_ (0.13), pointing toward a higher metallicity of the smaller clusters. The Gaussian width values are also comparable and are in the range 0.64–0.70 eV, being broader than those typically observed for bulk or surface metallic molybdenum. After oxygen exposure, Mo 3d core-level spectra were fitted using DFT-computed core levels. Computed core levels were used to construct the fitting function for the experimental spectra. Each fitting function is defined as the sum of a number of components. Particularly, in this case, the different components correspond to a different Mo valence families. Assuming that atomic valence corresponds to the oxidation state, atoms can be grouped into families based on the calculated valences. Specifically, the first family is composed of atoms with valences in the range 0.3–1.5e (denoted by Mo-1e), thus centered around 1. The second family corresponds to atoms with valences spanning from 1.5 to 2.5e (denoted by Mo-2e), and so on. Therefore, in principle, a Mo 3d spectrum can be fitted using a maximum of 7 components: one for the metallic atoms, and one corresponding to each Mo-valence family. The lineshape of these components, the Lorentzian and Gaussian widths and the asymmetry parameter, are constrained to be identical for all components. The background was modeled with a polynomial curve.

### Computational Methods

Theoretical simulations were performed using density functional theory (DFT). Geometry optimizations were performed by means of the Quickstep module of the CP2K package^50^ using periodic boundary conditions. The exchange–correlation energy was described by the Perdew–Burke–Ernzerhof (PBE) functional^69^ augmented with Grimme’s D3 dispersion correction.^70^ Core–valence interactions were treated by Goedecker–Teter–Hutter (GTH) pseudopotentials in combination with DZVP-MOLOPT basis sets. The plane-wave cutoff and relative cutoff were set to 400 and 55 Ry, respectively. The self-consistent field (SCF) cycle employed Fermi–Dirac smearing with an electronic temperature of 500 K and Broyden mixing for density convergence, with a threshold of 10^–6^. Geometry optimizations were carried out using the L-BFGS algorithm until the maximum atomic forces and displacement components were below 10^–3^ a.u. The same graphene/Ru(0001) system was used here as in previous works.^40^ This system is described by a slab of 4 layers of Ru in a 12 × 12 hexagonal supercell and a layer of 13 × 13 unit cells of graphene placed on top, with an overall number of 914 atoms, excluding Mo clusters. As this system was originally relaxed using VASP,^71^ prior to CP2K simulations, we ran it through CP2K relaxation again but did not find significant changes. Two bottom layers (288 atoms) were fixed during geometry optimization to mimic the bulk system underneath. Due to the very large size of the computational cell, the Brillouin zone sampling was restricted to the Γ point only in all our calculations.

Bader charges on atoms were calculated using the VASP code and CP2K-relaxed geometries. Positive gain corresponds to the excess of the electronic charge on the atom with respect to the neutral atom, while negative gain corresponds to the charge depletion.

Core-level BEs were also calculated using VASP (employing the method originally described in ref 72) and CP2K-relaxed geometries. The BE is adopted as the energy difference between the ground-state system and the one in which a 3d core electron localized on the Mo atom of interest is promoted to the valence band; subsequently, the Mo atom pseudopotential is also modified to reflect the presence of a core hole in it. Note that in the excited state, the electronic structure is driven to self-consistency, allowing for valence electron relaxation around the core hole. Using this approach, typical agreement between experiment and theory is normally better than 50 meV.^37,39,58,73–76^

Mo clusters were placed in the FCC valley of graphene as neutral. The strategy used to incorporate O atoms was based on their random distribution outside and inside the clusters, which is the only viable strategy for subsequent DFT calculations considering the immense size of our systems (more than 1000 atoms). Initially, at small O coverages, O atoms were placed at the exterior of the clusters prior to the full DFT relaxation. As the surface of the clusters became sufficiently populated, we tried to initially place additional O atoms in the cluster’s interior as well, and then the whole system was fully relaxed. Each time, we attempted to distribute O atoms as uniformly as possible across both the surface and inside the clusters. Details of the calculations of the energy barriers for oxygen molecule dissociation on a cluster and oxygen atom penetration into a cluster interior from the outside of the cluster are given in the Supporting Information.

## Supplementary Material







## Notes

Effect of photon irradiation on Mo 3d core-level spectra (Figure S1); structural models of Mo_13_O_65_ clusters investigated, in addition to the lowest-energy configuration (Figure S2); O–O distances distribution for Mo_13_O_n_ (Figure S3); computed energy barrier for an O atom for moving from cluster surface to cluster interior for Mo_13_O_30_ (Figure S4); Mo–Mo distances distribution as a function of oxygen density (Figure S5); variation of Mo–Mo bond lengths as a function of the oxygen density for Mo_6_ and Mo_13_ (Figure S6); O and Mo atoms charge gain as a function of O density (Figure S7); charge density difference isosurfaces for Mo_13_O_2_ supported on graphene and Mo_13_O_2_ supported on graphene/Ru(0001) (Figure S8); oxygen electron gain as a function of the oxygen–molybdenum bond lengths (Figure S9); minimum energy path for the dissociation of O_2_ molecule adsorbed on the Mo_13_ on graphene (Figure S10); top and side views of relaxed geometries of pristine Mo_6_ and Mo_13_ isomers (Figure S11); and mass spectrum of Mo^+^ nanoclusters (Figure S12) (PDF)
Charge density difference isosurfaces for Mo_10_ (Video 1) (MP4)
Charge density difference isosurfaces for Mo_13_ (Video 2) (MP4)
