# Supplementary material for: Photon-Driven Cluster Fluxionality: Breaking the Bulk Stoichiometry Ceiling in Subnanometric Molybdenum Oxides
Source: ACS Nano. 2026 Sep 8;20(37):25481–91. doi: 10.1021/acsnano.6c09355 (PMC13602921; doi:10.1021/acsnano.6c09355)
Supplement: Supplementary file 1 [file nn6c09355_si_001.pdf]

Supporting Information: Photon-Driven Cluster Fluxionality: Breaking the Bulk Stoichiometry Ceiling in Sub-Nanometric Molybdenum Oxides

Yao Wei[1,#], Deborah Perco[2,#], Alejandro Santana-Bonilla[1], Federico Loi[2,3], Paolo Lacovig[4], Silvano Lizzit[4], Lev Kantorovich[1] and Alessandro Baraldi[2,4,*]

[1] Theory and Simulation of Condensed Matter (TSCM), King's College London, London WC2R 2LS, UK

[2] Department of Physics, University of Trieste, Via Valerio 2, 34127 Trieste, Italy.

[3] J. Heyrovsky Institute of Physical Chemistry, Dolejškova 2155/73, 182 23 Prague, Czech Republic.

[4] Elettra - Sincrotrone Trieste, S.S. 14 Km 163.5, AREA Science Park, 34149 Basovizza-Trieste, Italy.

*Corresponding author: Alessandro Baraldi, alessandro.baraldi@elettra.eu

Keywords: fluxionality, nanoclusters, oxidation, molybdenum, oxygen activation, XPS, DFT

SUPPORTING INFORMATION FIGURES

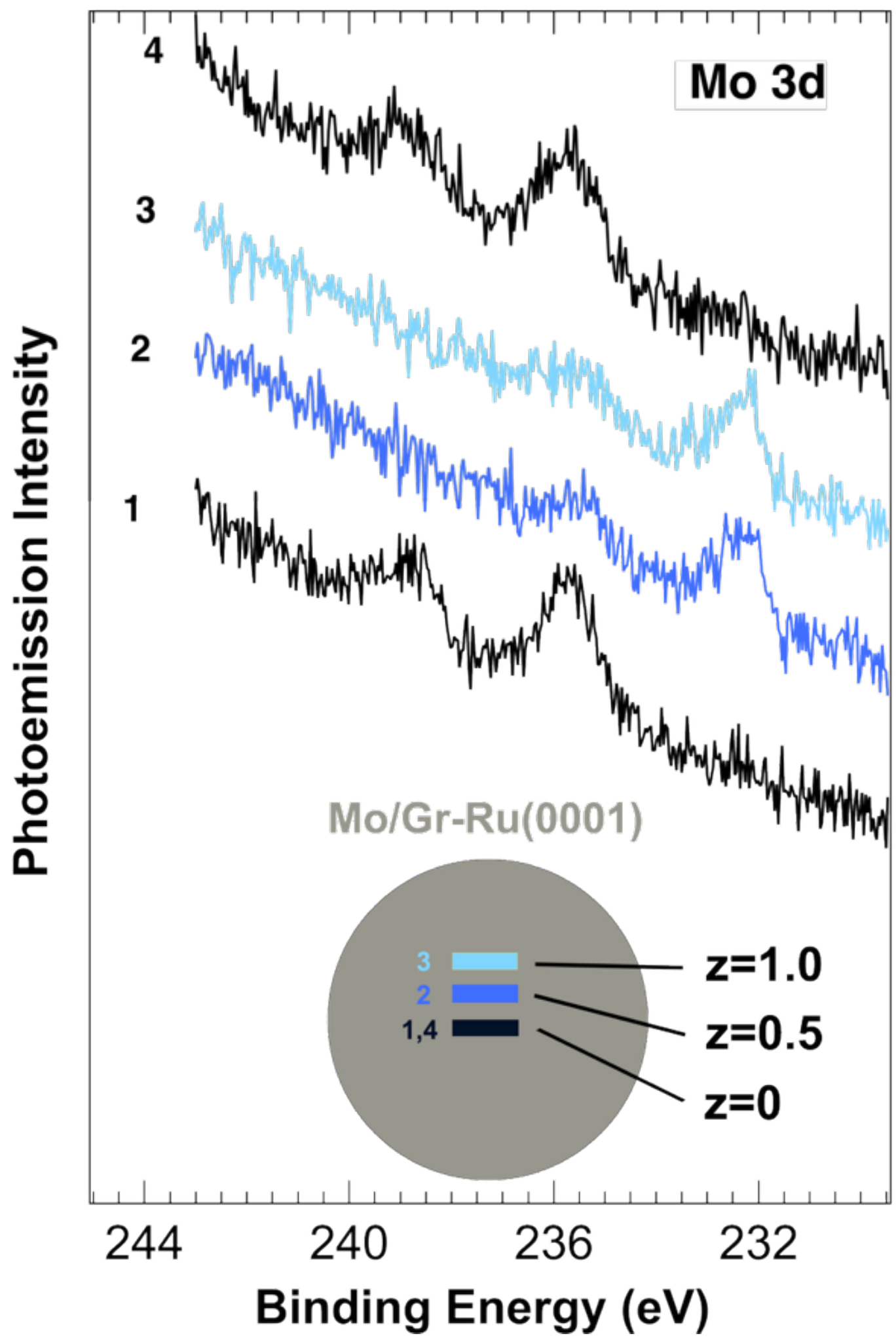

Figure S1. Mo 3d core level spectra measured after x-ray photon beam irradiation (black curves) and without exposure (coloured curves) in different part of the sample. Photon energy for irradiation and measurement was kept always equal to 320 eV.

Mo 3d spectra (hν=320 eV), acquired after exposure to 10 L of O_2 at 40 K above different regions of the sample, schematically represented in grey: (1) at a position previously exposed to X-rays for 20 minutes; (2) at a position displaced by 0.5 mm from the original location and not irradiated; (3) at a position displaced by 1 mm from the original location and not irradiated; and finally (4) again at the original irradiated position. The spectra were acquired using short acquisition times (60 seconds per spectrum) to minimize irradiation-induced effects on the sample. They reveal the transition from a metallic state, where the interaction of physisorbed O_2 molecules does not induce any relevant shifts in the measured Mo 3d

core electron binding energies, to an oxide state resulting from the photon-induced dissociation of the O-O bond and formation of Mo-O bonds.

The O_2 dissociation process is effective over a photon-energy range extending from 120 (the minimum photon energy available at the beamline) to 650 eV, corresponding to photon fluxes ranging from $9x10^{11}$ (120 eV) to $4.5x10^{11}$ (650 eV) photons s^{-1}, when the surface is exposed to X-ray for 20 minutes, with the X-ray photon beam spot size being of about 200 x 20 μm^2.

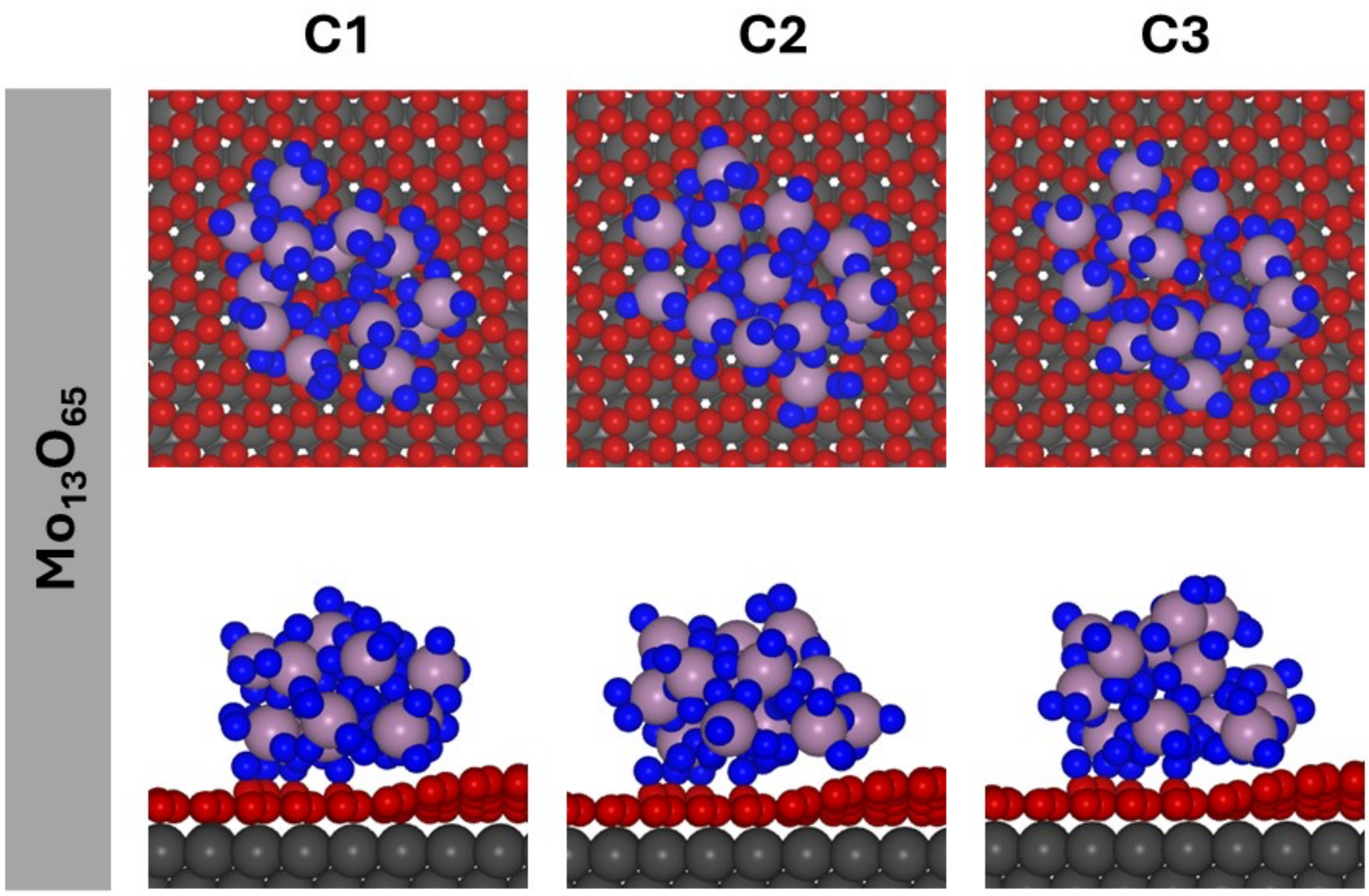

Figure S2. Structural model of different isomers of $Mo_{13}O_{65}$ clusters investigated, in addition to the lowest energy configuration reported in Fig. 2, to improve the statistical sampling used for calculating the Mo 3d core electron binding energies (upper panels: top view; lower panels: side view).

Figure S2 represents three different stable isomers of $Mo_{13}O_{65}$ obtained in our DFT calculations. These three structures were added into the statistical analysis of the computed Mo-core levels alongside all previously calculated geometries, showing a similar distribution of the BEs of Mo atoms, as discussed in the main text. This demonstrates that the distribution of the BEs of Mo atoms depends only weakly on the exact adsorption geometry of O atoms.

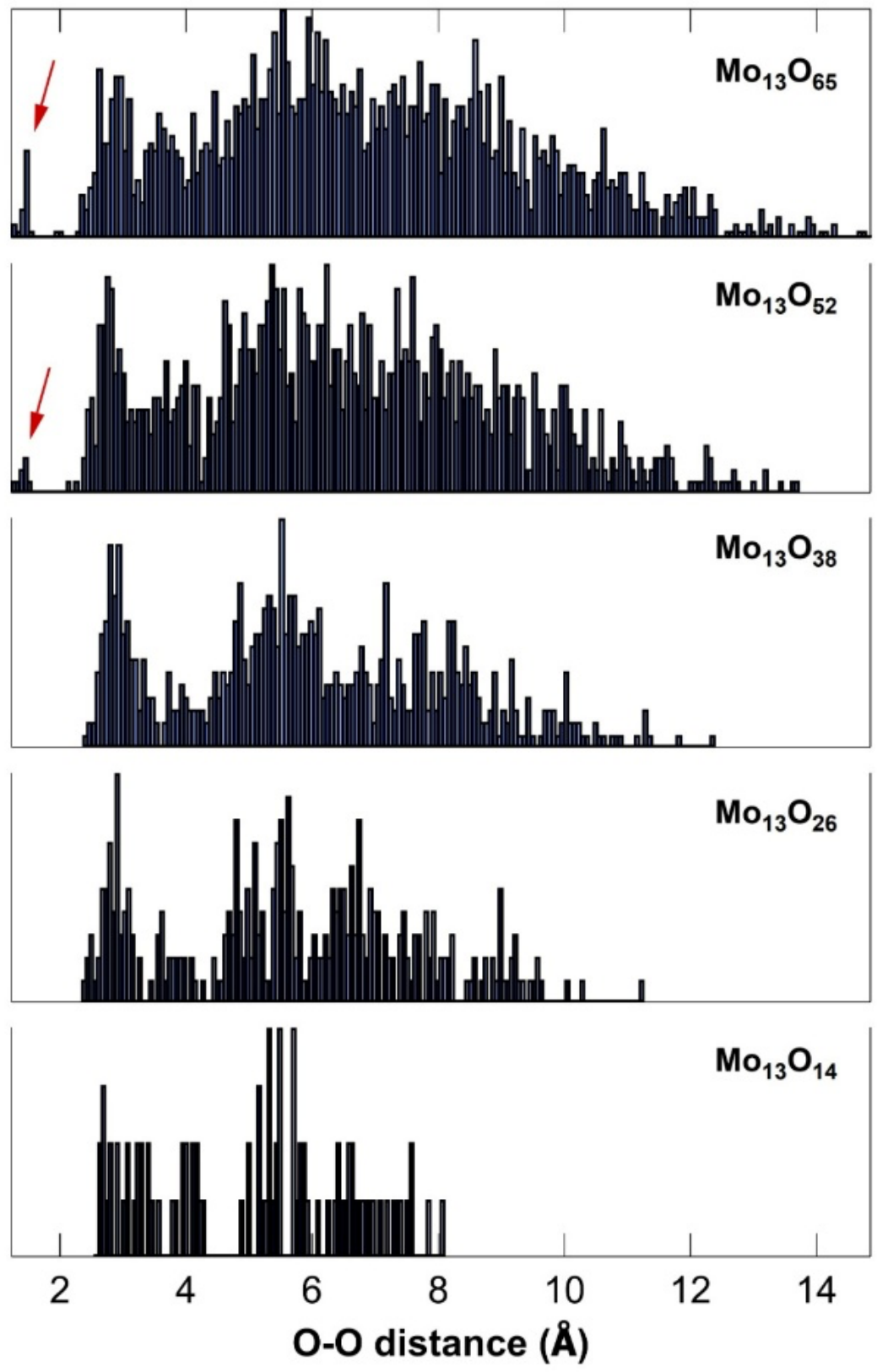

Figure S3. O-O distances distribution for $Mo_{13}O_n$ (n=14, 26, 38, 52, 65).

Figure S3 shows the distribution of O-O distances in Mo_{13} clusters as the oxygen density increases. Only for Mo:O ratio lower than 1:3, the O-O distances in the range 1.3-1.4 Å are found, which are typical of peroxo and superoxo species (indicated by arrows). However, these species represent only 1% of the oxygen distances in the simulated nanoclusters.

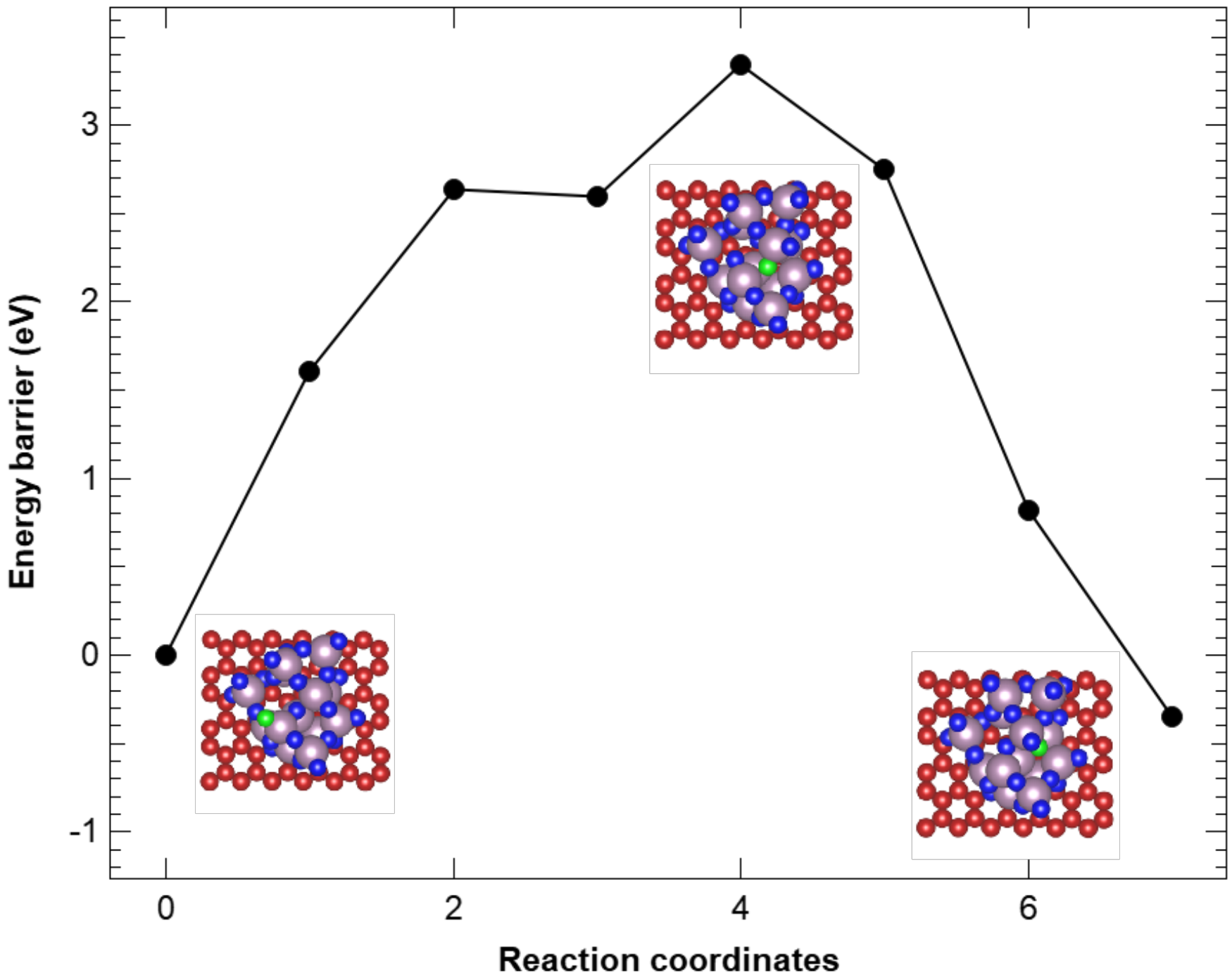

Figure S4. Computed energy barrier for an O atom for moving from cluster surface to cluster interior for $Mo_{13}O_{30}$

Figure S4 represents the NEB computed energy barrier for an O atom to move from the cluster surface to its interior. The high value of this barrier, i.e. 3.69 eV, suggests that this process requires a significant amount of energy, since it involves a significant rearrangement of the atoms in the cluster. The required energy comes from the irradiation and subsequent secondary electrons providing the necessary vibrational excitations facilitating the rearrangements.

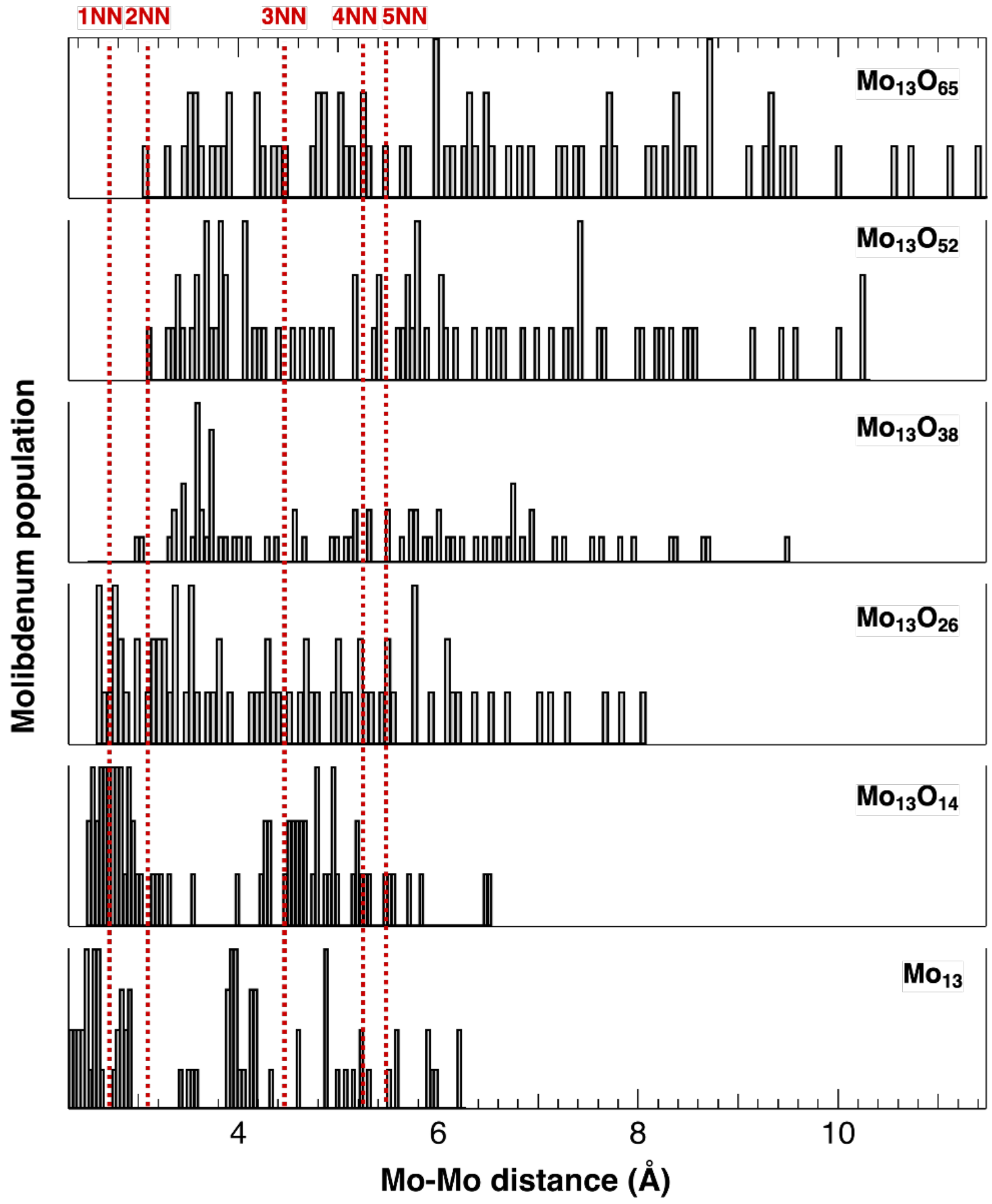

Figure S5. Mo-Mo distances distribution as a function of oxygen density.

Figure S5 displays the Mo-Mo distances distribution as a function of oxygen density. The evolution of the Mo-Mo bond-length distribution clearly shows that this quantity increases with oxygen coverage, evolving from values comparable to the nearest-neighbour distance in bulk molybdenum (2.74 Å) to values that even exceed the second nearest-neighbour distance (3.17 Å) there. These and further nearest-neighbour distances are indicated by vertical dashed lines.

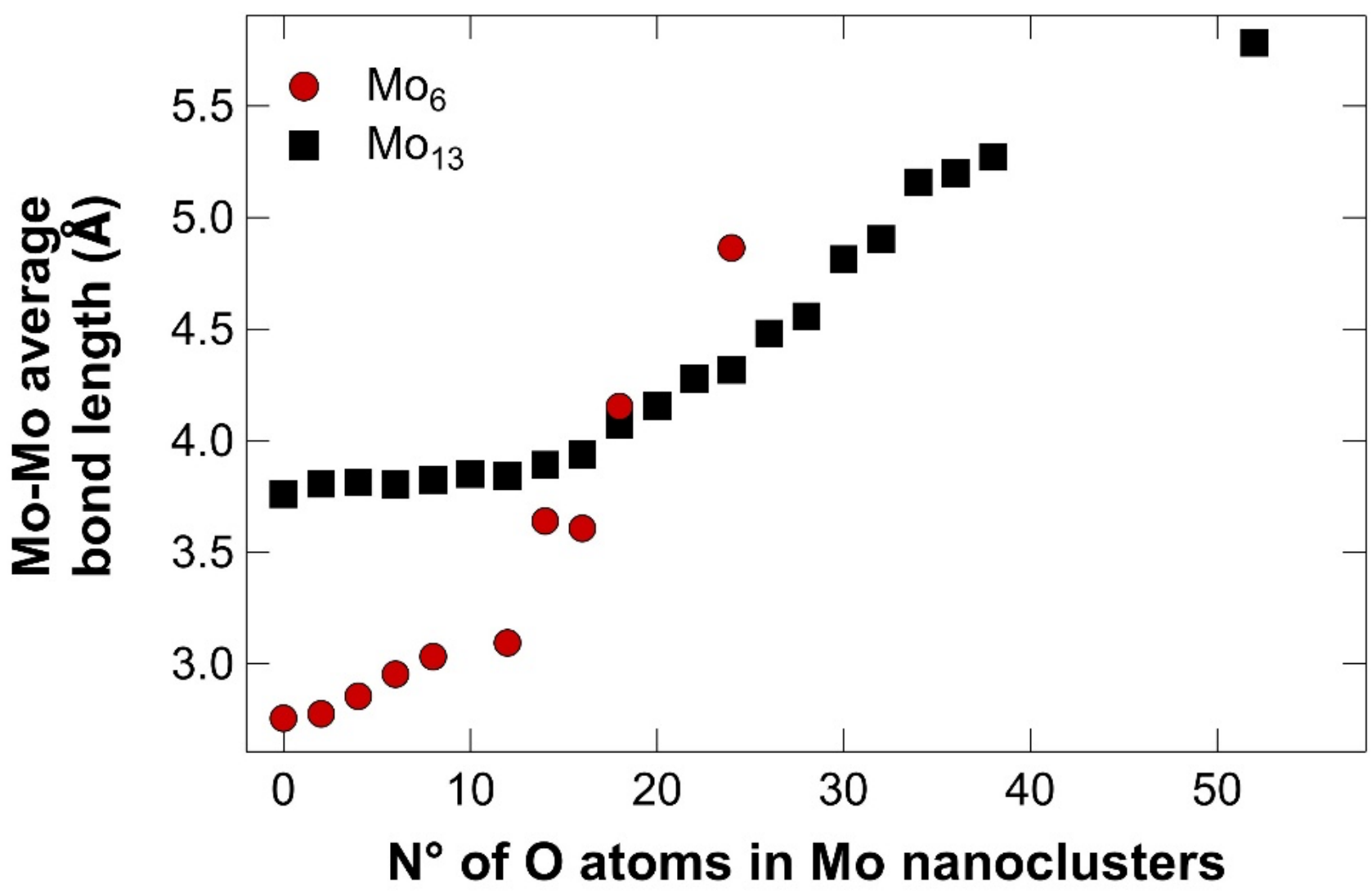

Figure S6. Variation of Mo-Mo bond lengths as a function of the oxygen density in the two clusters.

Figure S6 shows the evolution of the average Mo–Mo distances in Mo_6 and Mo_{13} nanoclusters as a function of the number of incorporated oxygen atoms, providing a quantitative measure of the structural response of the clusters to oxidation. For both clusters, the average Mo–Mo distance increases monotonically with increasing oxygen content, reflecting the progressive weakening of metal–metal bonding due to charge transfer from Mo atoms to the electronegative oxygen atoms and the formation of Mo–O bonds. This expansion is a direct structural signature of oxidation and accompanies the gradual transition from metallic bonding towards oxide-like local environments. A marked size dependence is observed. In the case of Mo_6, the increase in Mo–Mo bond length is significantly steeper than for Mo_{13} at comparable oxygen densities. This indicates that the smaller cluster undergoes a much stronger structural distortion upon oxidation. Owing to its limited number of Mo–Mo bonds and reduced structural rigidity, oxygen incorporation in Mo_6 more efficiently disrupts the metallic framework, leading to a rapid expansion of the cluster. In contrast, Mo_{13} exhibits a more gradual increase of the Mo–Mo bond length over a wider range of oxygen coverages. The larger number of Mo atoms and the more disordered three-dimensional geometry allow Mo_{13} to better accommodate oxygen atoms while partially preserving Mo–Mo connectivity. As a result, structural expansion is distributed more evenly across the cluster. At high oxygen densities, both clusters reach Mo–Mo distances substantially larger than those characteristic of

the pristine metallic clusters, consistent with the emergence of highly oxidized configurations dominated by Mo–O bonding. This pronounced lattice expansion supports the conclusion drawn in the main text that oxidation induces a fundamental reorganization of the cluster framework, particularly in smaller clusters.

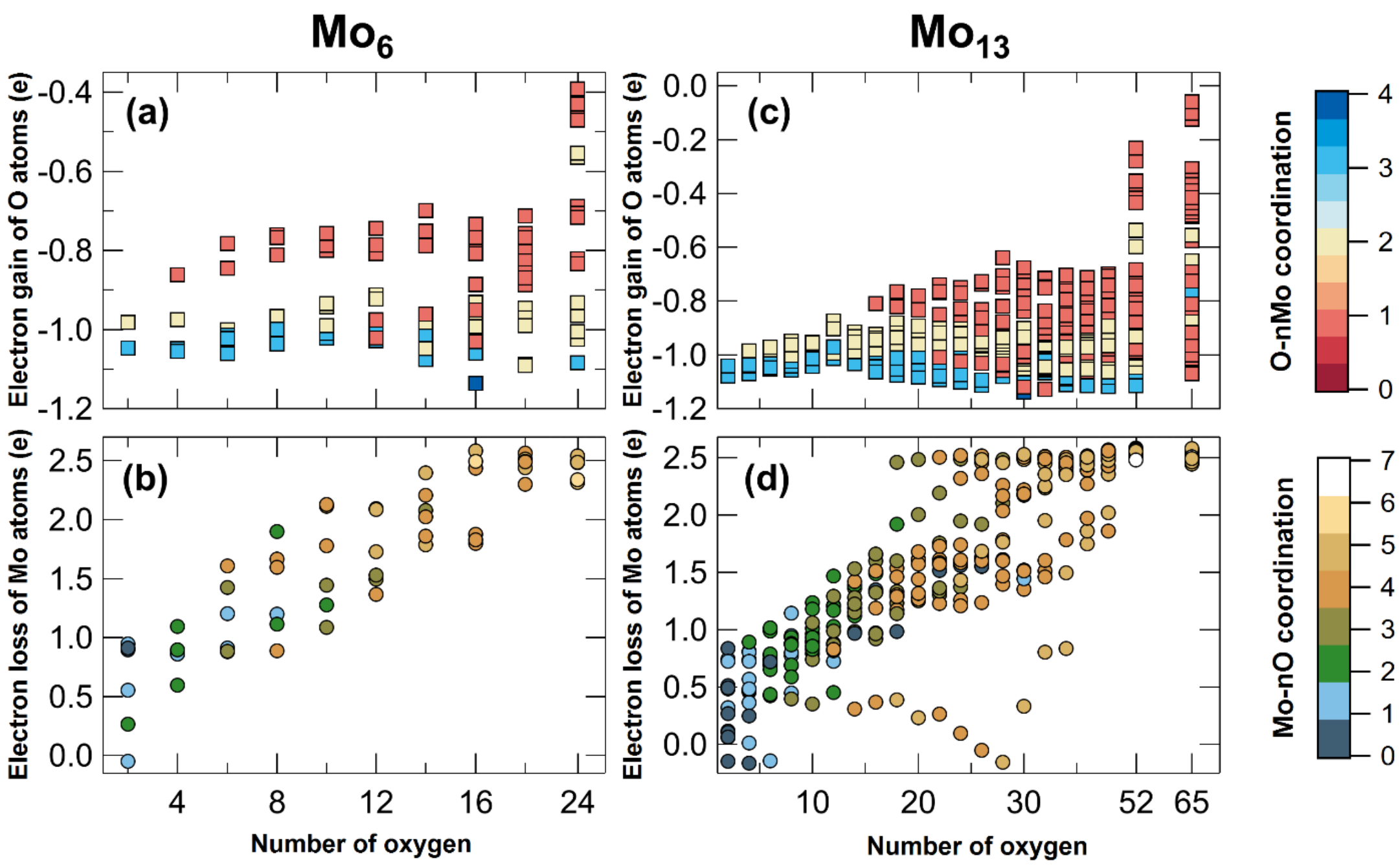

Figure S7. Upper panels: O atoms charge gain as a function of O density for Mo_6O_x (a) and $Mo_{13}O_x$ (c) clusters. Different colours correspond to different coordination of the O atoms by Mo atoms. Lower panels: Mo atoms charge gain as a function of O density for Mo_6O_x (b) and $Mo_{13}O_x$ (d) clusters. Different colours correspond to different coordination of the Mo atoms by O atoms.

Figure S7(a) reports the Bader charge gain of individual oxygen atoms in Mo_6O_{2x} clusters as a function of the total oxygen content, with different colours indicating distinct O coordination environments. At low oxygen densities, oxygen atoms exhibit a narrow charge gain distribution centred around ~1.0 e, reflecting predominantly multi-coordinated, bulk-like bonding motifs. With increasing oxygen density, the charge distribution broadens substantially, extending down to ~0.4–0.6 e at the highest oxygen

contents. This evolution signals the emergence of diverse local oxygen environments, including low-coordinated and weakly bound species, for which charge transfer from Mo is reduced. Figure S7(b) shows the corresponding Bader charge depletion of Mo atoms in Mo_6O_{2x} clusters. At low oxygen densities, Mo atoms lose between −0.5 e and −1.0 e, indicating moderate oxidation and the persistence of partially metallic Mo–Mo bonding. As the oxygen content increases, Mo charge depletion becomes stronger and more heterogeneous, reflecting the progressive involvement of additional Mo atoms in Mo–O bonding. At high oxygen densities, the Mo charge distribution narrows and saturates at approximately −2.5 e, indicating that Mo atoms reach a maximal effective oxidation level. Beyond this point, further oxygen incorporation occurs without a proportional increase in charge donation from Mo. Figure S7(c) presents the oxygen charge gain in $Mo_{13}O_{2x}$ clusters. Compared to Mo_6O_{2x}, a narrow, bulk-like charge distribution persists over a wider range of oxygen densities, consistent with the larger structural flexibility and higher number of available Mo coordination sites. At high oxygen densities, however, the distribution broadens markedly, with a significant fraction of oxygen atoms exhibiting very low electron gain (<0.3 e). These species correspond to weakly bound or peripheral oxygen atoms that are not directly coordinated to Mo.

Figure S7(d) shows the Mo charge depletion in $Mo_{13}O_{2x}$ clusters. The oxidation process proceeds more gradually than in Mo_6O_{2x}, with Mo atoms progressively donating charge as oxygen density increases. As in the smaller cluster, the Mo charge depletion saturates at approximately −2.5e at high oxygen contents, indicating a size-independent upper limit to the effective oxidation level of individual Mo atoms. Taken together, Figure S7 demonstrates a decoupling between oxygen incorporation and charge transfer at high oxygen densities. While the electronic charge donated by Mo atoms saturates, the electronic states of oxygen atoms continue to diversify, reflecting the formation of non-bulk-like bonding motifs. This behaviour underlines the breakdown of stoichiometric oxidation concepts at the sub-nanometric scale and supports the valence-based interpretation adopted in the main text.

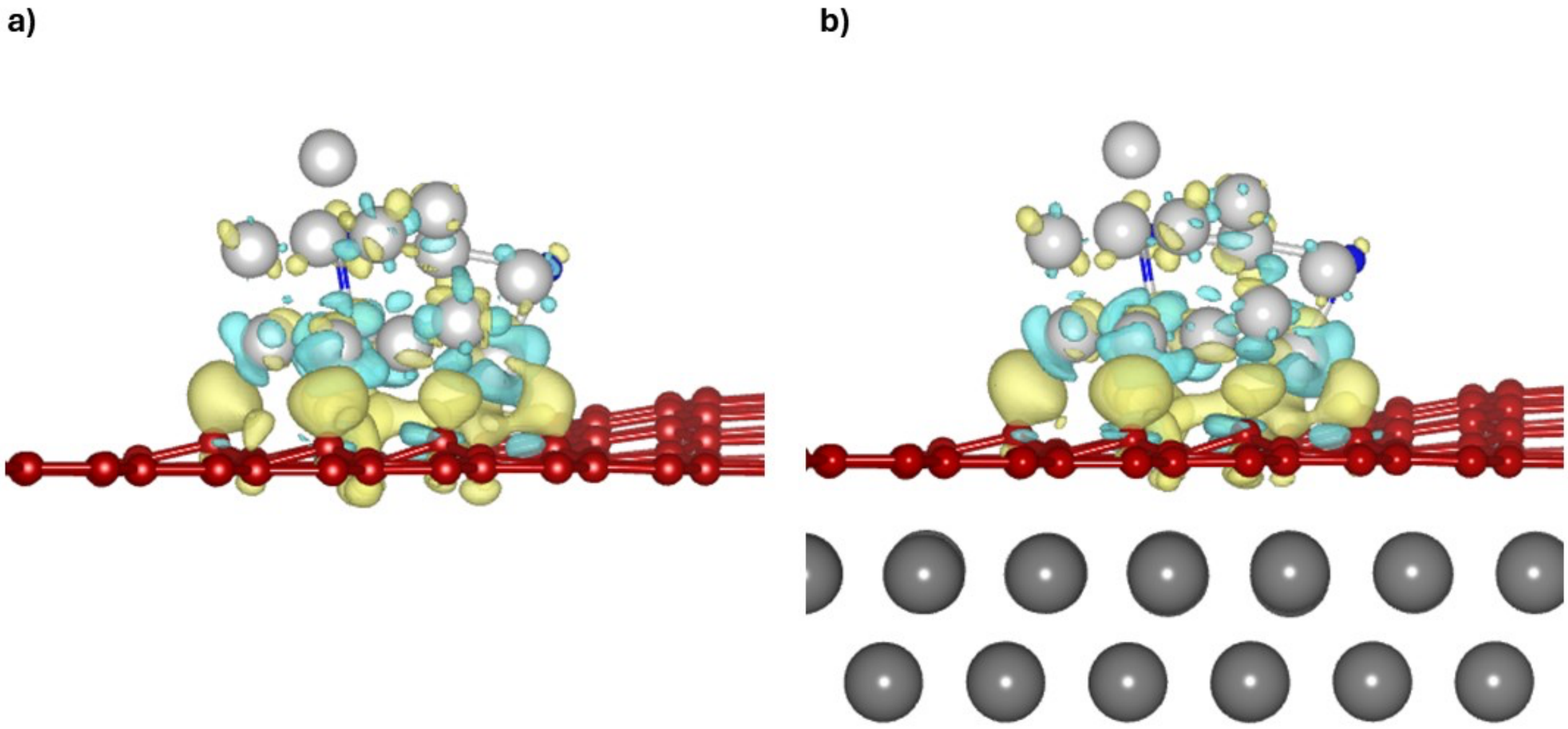

Figure S8. Visualization of charge density difference isosurfaces (with green at +0.007 e/Å (depletion) and yellow at -0.007 e/Å (excess)) for (a) $Mo_{13}O_2$ supported on graphene and (b) $Mo_{13}O_2$ supported on graphene/Ru(0001).

Figure S8 shows the distribution of the charge densities difference for two systems: the cluster on graphene (left) and on graphene/Ru interface. The charge density difference was computed by subtracting the electronic densities of the individual systems (the cluster and the surface) from the density of the combined relaxed system, keeping the same geometry as in the combined system for the individual components. It is seen that there is a clear charge transfer from the Mo cluster to the graphene layer. In contrast, the contribution of the Ru substrate is relatively small, leading only to a partial compensation of the electron withdrawal induced by graphene. Moreover, the charge transfer strongly depends on the distance between the metal atoms and the substrate: the electron depletion is significantly more pronounced for the bottom-layer Mo atoms, i.e., those directly facing the graphene, than for the atoms located in the upper part of the cluster.

Two movies of the charge difference for the (a) and (b) structures are also included in the submission (Video 1 and Video 2).

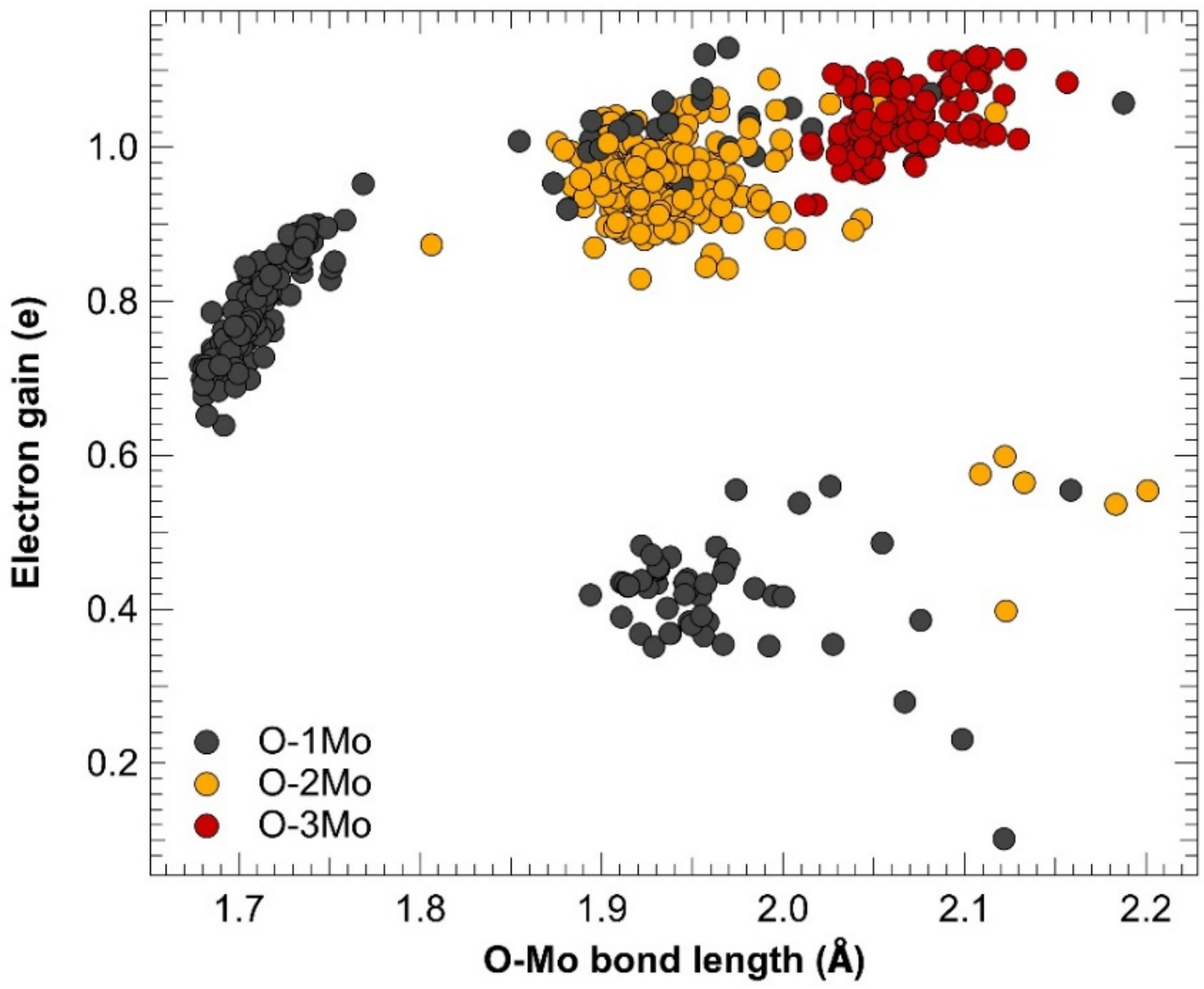

Figure S9. Oxygen electron gain as a function of the oxygen-molybdenum bond lengths. Different colours are used to identify different oxygen coordination with Mo.

A clear dependence of the electronic charge transfer on both the O–Mo bond length and the oxygen coordination is observed. Oxygen atoms coordinated to a single Mo atom (O–1Mo) exhibit the shortest O–Mo bond lengths, clustered around ~1.7–1.8 Å, and the largest electron gain, typically in the range of 0.7–0.9 e. These short bond lengths and high electron transfer are characteristic of terminal oxo species (Mo=O), consistent with the formation of double bonds and strong covalent Mo–O interactions. Oxygen atoms coordinated to two Mo atoms (O–2Mo) populate an intermediate region of bond lengths, centred around ~1.9–2.0 Å, and show a broader distribution of electron gain, typically around 0.9–1.0 e. This reflects the shared nature of the bonding, where the electronic charge transferred from Mo is distributed between multiple oxygens, leading to slightly elongated bonds and reduced localization compared to terminal oxo ligands. In contrast, oxygen atoms coordinated to three Mo atoms (O–3Mo) are associated with the longest O–Mo bond lengths, extending beyond 2.0 Å, and exhibit the largest average electron gain, approaching or exceeding 1.0 e. These species correspond to highly coordinated, bridging oxygen environments typical of bulk-like oxide motifs, where the cumulative charge transfer from several

Mo atoms compensates for the longer bond lengths. The separation of the data into distinct clusters in the bond-length–charge plane demonstrates that oxygen coordination, rather than bond length alone, is a key factor controlling the local electronic structure of oxygen atoms in oxidized Mo nanoclusters. The emergence of a substantial population of O–1Mo species with short bond lengths and well-defined electron gain provides direct evidence for the stabilization of terminal oxo ligands at high oxygen densities, supporting the molecular-like oxidation motifs discussed in the main text.

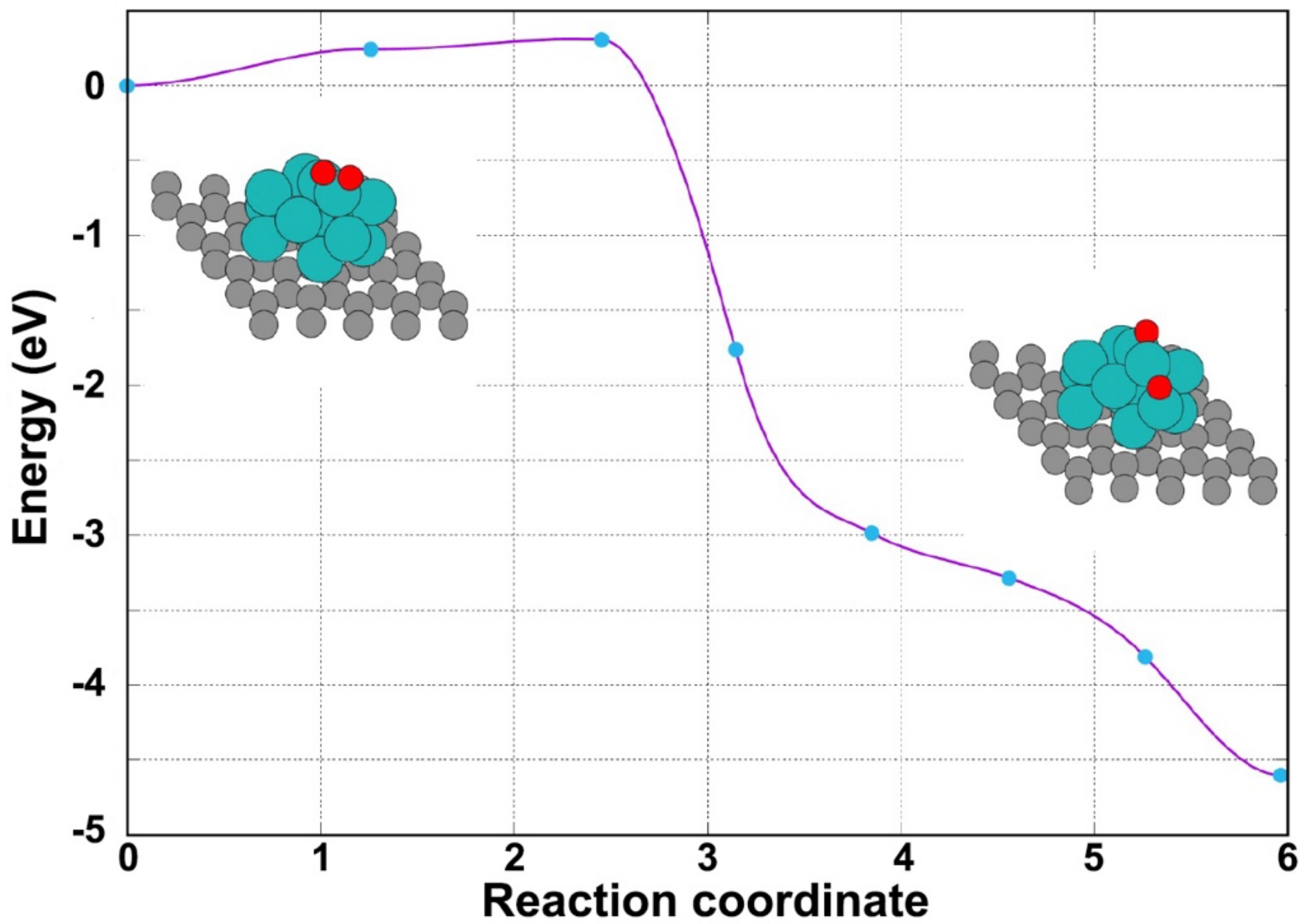

Figure S10. Minimum energy path (MEP) for the dissociation of O_2 molecule adsorbed on the Mo_{13} cluster placed on the layer of graphene. The initial (left) and final (right) geometries of the dissociation path are also shown.

The calculation of the energy barrier for the dissociation of the O_2 molecule adsorbed on the Mo_{13} cluster was done using the Nudged Elastic Band (NEB) method [1,2] as implemented in VASP. In these calculations 7 images were used. To make the calculation feasible, we removed the Ru substrate and fixed atomic positions in the graphene layer as well as of the lowest Mo atoms bound to graphene. Since the spin states of the initial and final geometries were different, we enabled optimisation of magnetic

moments on all O and Mo atoms during the NEB calculations. The results of the calculation are shown in Fig. S10. The barrier is found to be 0.25 eV.

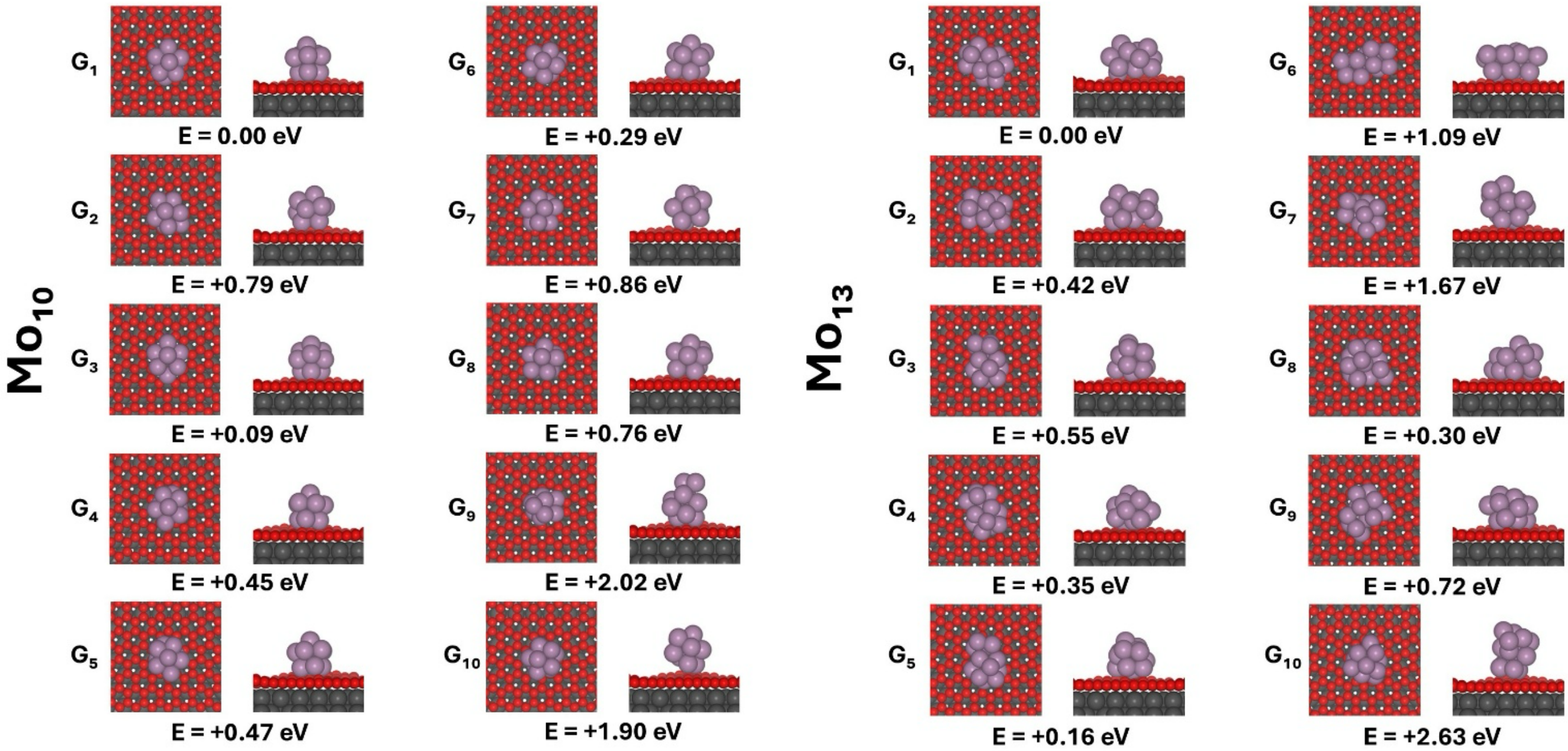

Figure S11. Top and side views of relaxed geometries of pristine Mo_6 and Mo_{13} isomers.

Figure S11 shows the DFT-computed isomers (G_1-G_{10}) for Mo_6 and Mo_{13}, with energies referenced to the lowest-energy structure. Notably, most geometries fall within 1 eV of the most stable isomer, supporting our conclusion that a broad range of configurations can be accessed through secondary electron-induced deformations.

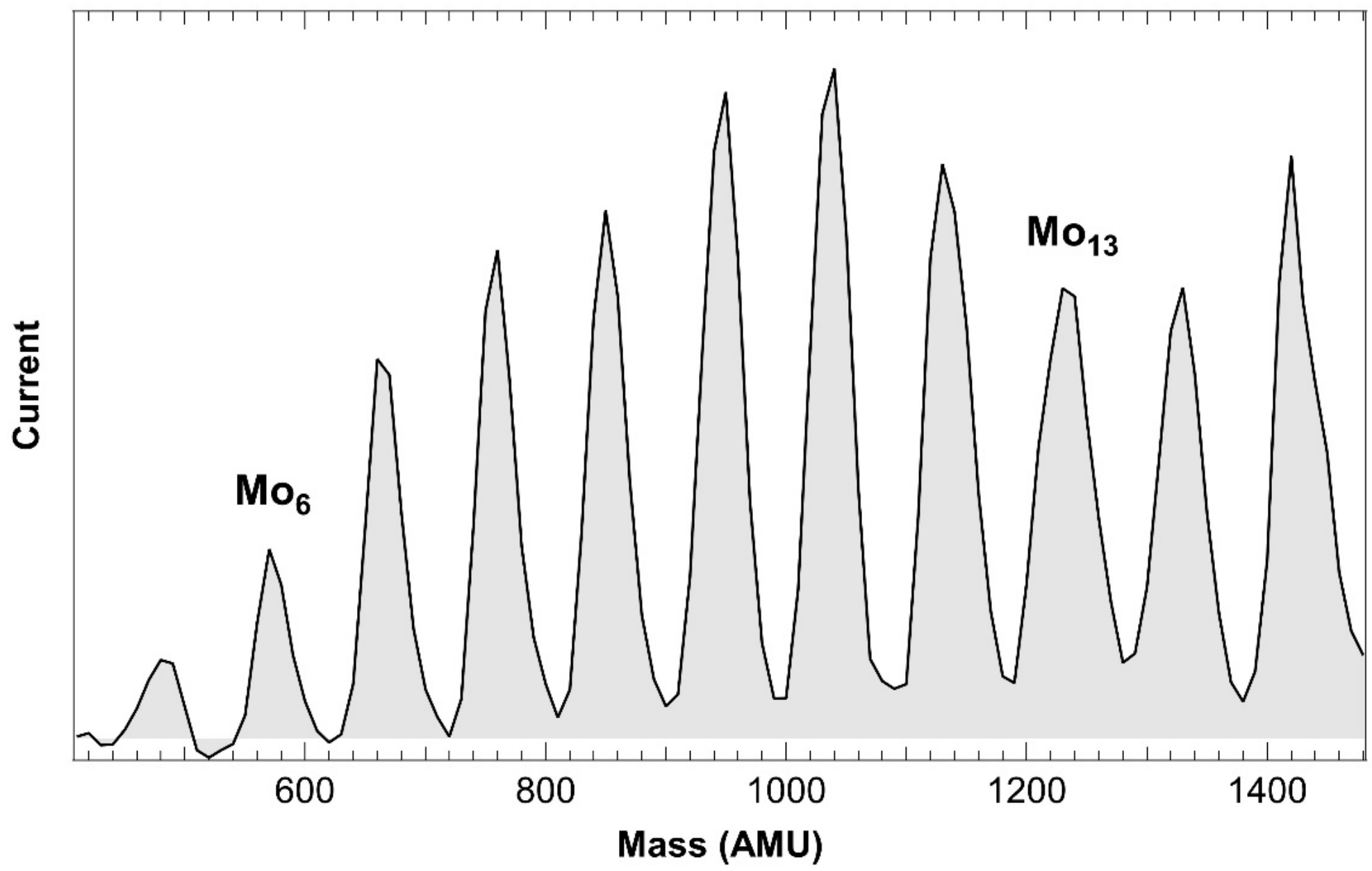

Figure S12. Mass spectrum of Mo^+ nanoclusters generated by ENAC.

Figure S12 displays a representative mass spectrum of Mo^+ nanoclusters produced by the ENAC (Exact Number of Atoms in Each Cluster) source prior to deposition. The spectrum exhibits a series of well-resolved peaks corresponding to molybdenum clusters with different numbers of atoms, demonstrating the ability of the source to generate clusters with precise atomicity. Distinct peaks corresponding to Mo_6^+ and Mo_{13}^+ clusters are clearly identified, confirming the availability of these specific cluster sizes for subsequent mass selection and deposition. The narrow width of the peaks indicates good mass resolution and minimal overlap between adjacent cluster sizes, ensuring that the deposited clusters are well defined in terms of composition. The intensity modulation across the spectrum reflects the relative abundance of clusters of different sizes generated under the experimental conditions but also reflects which mass to charge ratio is better transported using a defined set of parameters. Importantly, the clear separation between neighbouring mass peaks allows for efficient mass filtering using the quadrupole mass analyser, enabling the deposition of size-selected Mo_6 and Mo_{13} clusters with negligible contamination from other cluster sizes. This mass spectrum confirms the reliability of the ENAC source in producing and selecting molybdenum nanoclusters with atomic precision, which is essential for establishing a direct correlation

between cluster size, oxidation behaviour, and the electronic and structural properties discussed in the main text.

[1] Jónsson, H.; Mills, G.; Jacobsen, K. W. Nudged elastic band method for finding minimum energy paths of transitions. In *Classical and Quantum Dynamics in Condensed Phase Simulations*, B. J. Berne, G. Ciccotti, and D. F. Coker (Eds.), World Scientific, **1998**; pp 385-404.

[2] Henkelman, G.; Uberuaga, B. P.; Jónsson, H. A climbing image nudged elastic band method for finding saddle points and minimum energy paths. *J. Chem. Phys.* **2000,** 113, 9901-9904.
